# Trichopolydesmidae from Cameroon, 1: The genus *Hemisphaeroparia* Schubart, 1955. With a genus-level reclassification of Afrotropical genera of the family (Diplopoda, Polydesmida)

**DOI:** 10.3897/zookeys.785.27422

**Published:** 2018-09-19

**Authors:** S. I. Golovatch, A. R. Nzoko Fiemapong, J. L. Tamesse, J.-P. Mauriès, D. VandenSpiegel

**Affiliations:** 1 Institute for Problems of Ecology and Evolution, Russian Academy of Sciences, Moscow, Russia; 2 Laboratoire de Zoologie, Université Yaoundé 1, BP812, Cameroun; 3 Laboratory of Zoology, Higher Teacher’s Training College, University of Yaounde I, P.O.Box 47, Yaounde, Cameroon; 4 Muséum national d’Histoire naturelle, Paris, France; 5 Musée Royal de l’Afrique centrale, Tervuren, Belgique

**Keywords:** Afrotropical, Cameroon, millipede, new combination, new species, new status. new synonymy, review, taxonomy Trichopolydesmidae

## Abstract

In addition to one of the two species of Trichopolydesmidae hitherto recorded from Cameroon, *Polydesmusintegratus* Porat, 1894, which is revised based on type material and shown to represent the genus *Hemisphaeroparia* Schubart, 1955, **comb. n.**, 12 new species from the same genus are described from that country: *H.zamakoe***sp. n.**, *H.bangoulap***sp. n.**, *H.spiniger***sp. n.**, *H.ongot***sp. n.**, *H.digitifer***sp. n.**, *H.parva***sp. n.**, *H.fusca***sp. n.**, *H.bonakanda***sp. n.**, *H.bamboutos***sp. n.**, *H.subfalcata***sp. n.**, *H.falcata***sp. n.** and *H.mouanko***sp. n.** A key to all 13 species (of *Hemisphaeroparia*) known to occur in Cameroon is presented, and their distributions are mapped. All ten recognizable (but excluding two dubious) Afrotropical genera or subgenera of Trichopolydesmidae are rediagnosed and reclassified, based both on their type species and a presumed scenario of gonopodal evolution. As a result, the number of accepted genera is reduced to five: *Sphaeroparia* Attems, 1909 (= *Megaloparia* Brolemann, 1920), *Physetoparia* Brolemann, 1920 (= *Elgonicola* Attems, 1939, **syn. n.**, = *Mabocus* Chamberlin, 1951, **syn. n.**, = *Heterosphaeroparia* Schubart, 1955, **syn. n.**}, *Eburodesmus* Schubart, 1955, *Mecistoparia* Brolemann, 1926 (= *Dendrobrachypus* Verhoeff, 1941, **syn. n.**), and *Hemisphaeroparia*.

## Introduction

The millipede family Trichopolydesmidae, not long ago largely referred to as Fuhrmannodesmidae, a group that mainly included tropical taxa (e.g., Hoffman 1980, Golovatch 1994, Mauriès and Heymer 1996) as opposed to the originally Mediterranean Trichopolydesmidae, presently contains ca. 140 species from ca. 75 genera, mainly across the Northern Hemisphere, both temperate and, especially, tropical (Golovatch 2013, Golovatch and Enghoff 2015). Even after the resurrection of the small and mainly Nearctic family Macrosternodesmidae from synonymy to Trichopolydesmidae, as recently proposed by Shear and Reddell (2017), the diversity of trichopolydesmids in the restricted sense remains impressive.

Apparently because of the small to very small bodies (3–20 mm long), regional tropical faunas of Trichopolydesmidae are particularly poorly known. This fully applies to the Afrotropical realm which, based on the latest review of the mainland fauna (Mauriès and Heymer 1996) and updated here using the available information about adjacent insular faunas (Golovatch and Korsós 1992, Mauriès and Geoffroy 1999, Golovatch and Gerlach 2010), contains only two accepted genera (*Bactrodesmus* Cook, 1896 and *Sphaeroparia* Attems, 1909) and ca. 40 species. Quite a few of these species still remain obscure, e.g., *Sphaeroparia*? sp. from the Seychelles (Golovatch and Korsós 1992, Golovatch and Gerlach 2010) or several *Mauritacantha* spp. from the Reunion (= Mauritius) Island (Mauriès and Geoffroy 1999). The same also concerns *Polydesmusintegratus* Porat, 1894 and *P.parvulus* Porat, 1894, both from unknown localities and both representing the only species of Trichopolydesmidae hitherto reported from Cameroon (Porat 1894).

The present paper puts on record most of a rich fresh collection of Trichopolydesmidae from Cameroon, provides the results of a revision of the sole male type of *P.integratus* and thus seriously updates the fauna of this family not only in the country, but in entire Africa. Unfortunately, the type series of *P.parvulus*, which also contained male material (Porat 1894), remains unavailable for restudy, apparently being misplaced in the Stockholm Museum. A new generic classification of the Afrotropical trichopolydesmids is also proposed, a key to and a map of all trichopolydesmids currently known to occur in Cameroon are also given.

## Material and methods

Most of the material treated here derives from the collection of the Musée Royal de l’Afrique Centrale (MRAC), Tervuren, Belgium, with only a few duplicates retained for the collections of the University of Yaounde 1 (UY1) and the second author (ARNF), Cameroon or donated to the Zoological Museum, State University of Moscow (ZMUM), Russia, as indicated below. The samples are stored in 70% ethanol. Specimens for scanning electron microscopy (SEM) were air-dried, mounted on aluminium stubs, coated with gold and studied using a JEOL JSM-6480LV scanning electron microscope. The colour pictures were taken using the focus stacking setup described by Brecko et al. (2014). Canon EOS Utility software was used to control the camera. Zerene Stacker was applied for stacking the individual pictures into one ‘stacked image’.

The abbreviations used to denote gonopodal structures are explained directly in the text and figure captions.

## A generic reclassification of Afrotropical Trichopolydesmidae

The high number of generic categories (12 genera or subgenera, see below) to adopt some 35 species recorded throughout continental tropical Africa (Mauriès and Heymer 1996) is likely evidence of the poor state of the art. One more genus, *Mauritacantha* Verhoeff, 1939, from Mauritius, remains invalid as no proper typification has ever been done (Verhoeff 1939). The suggestion by Mauriès and Geoffroy (1999) of *Mauritacanthalawrencei* Verhoeff, 1939 as a potential type species of this genus is not a valid act of typification and is thus to be ignored.

**Figure 1.**
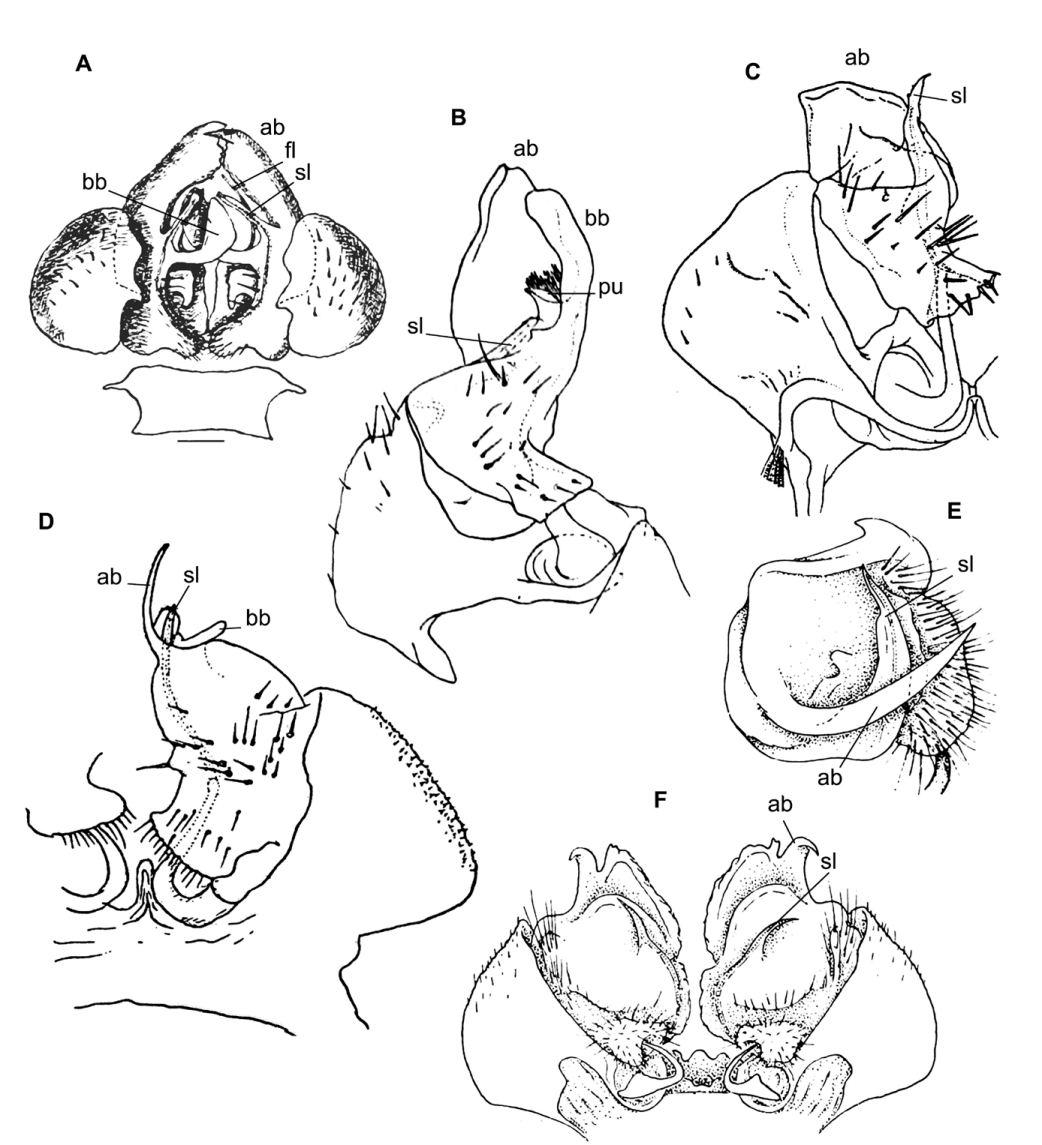
Gonopods of the type species of some African Trichopolydesmidae. **A***Sphaeropariaminuta* Attems, 1909 (after Attems 1909) **B**Sphaeroporia (Megaloparia) lignivora Brolemann, 1920 (after Brolemann 1920) **C**Sphaeroparia (Physetoparia) imbecilla Brolemann, 1920 (after Brolemann 1920) **D***Elgonicolajeanneli* Attems, 1939 (after Attems 1939) **E, F***Mabocusgranulifer* Chamberlin, 1951 (after Kraus 1958). Reproduced not to scale. Abbreviations: **ab** apical branch of telopodite, **bb** basal branch of telopodite, **fl** flagellum, **lo** lobe, **sl** solenomere, **pu** pulvillus.

**Figure 2.**
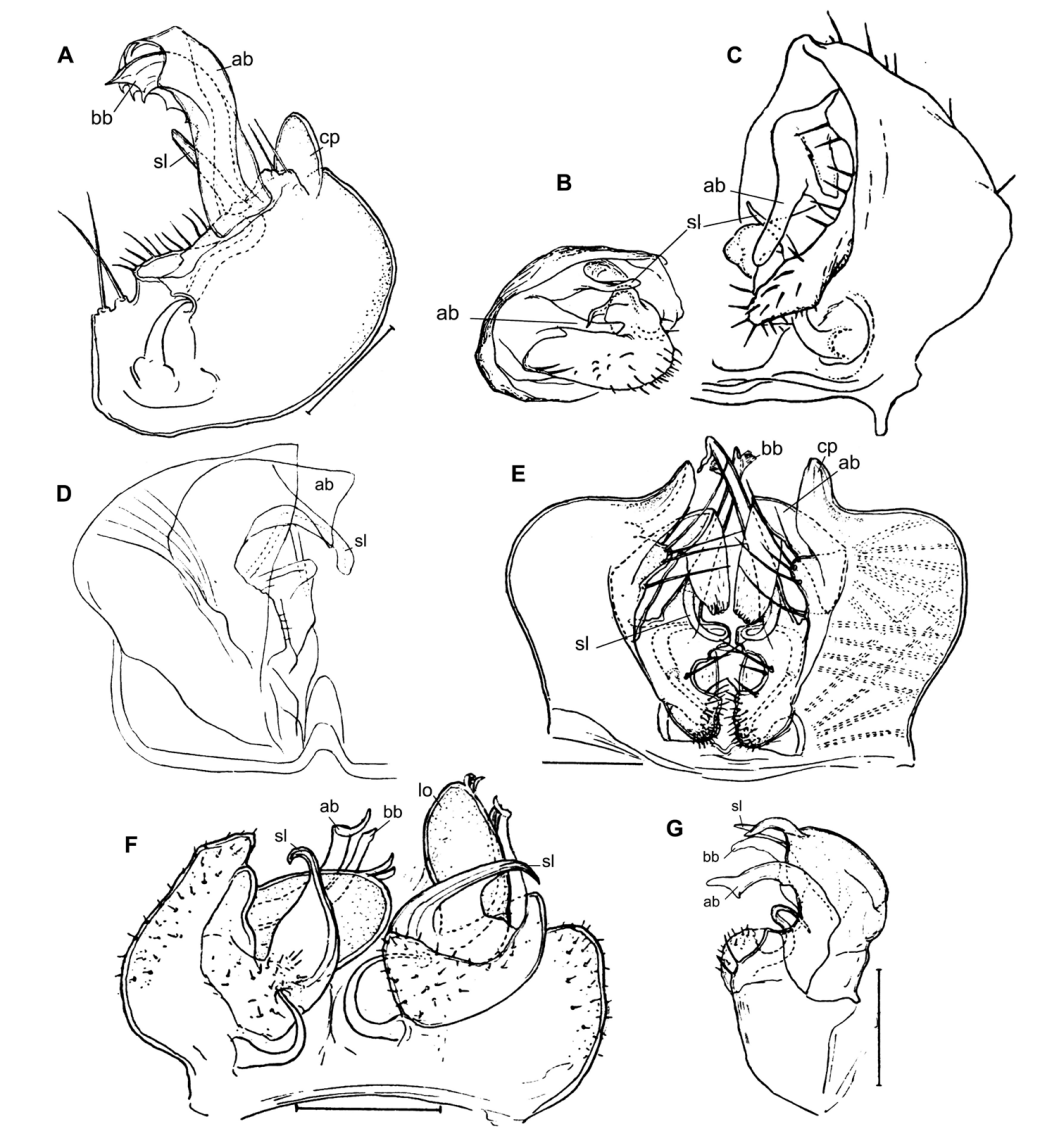
Gonopods of the type species of some African Trichopolydesmidae. **A***Heterosphaeropariavilliersi* Schubart, 1955 (after Schubart 1955) **B, C***Mecistoparialophotocrania* Brolemann, 1926 (after Brolemann 1926) **D***Dendrobrachypuspusillus* Verhoeff, 1941 (after Verhoeff 1941) **E***Eburodesmuserectus* Schubart, 1955 (after Schubart 1955) **F, G***Hemisphaeropariacumbula* Schubart, 1955 (after Schubart 1955). Reproduced not to scale. Abbreviations: **ab** apical branch of telopodite, **bb** basal branch of telopodite, **cp** coxal process, **lo** lobe, **sl** solenomere.

The following nomenclaturally available generic or subgeneric names have been suggested to accommodate the Afrotropical Trichopolydesmidae, arranged below in alphabetic order and followed by their brief descriptions and gonopodal characters (Figs 1, 2), as well as provenance, all this derived from the original descriptions of their type species alone.

### Bactrodesmus

Taxon classificationAnimaliaPolydesmidaTrichopolydesmidae

Cook, 1896

#### Type-species.

*Bactrodesmusclaviger* Cook, 1896, by subsequent monotypy; Liberia.

The genus was first proposed as a *nomen nudum* (Cook 1896a), but then properly typified (Cook 1896b). The sole useful information contained in the original description of *B.claviger* concerns its small size (7 mm long, 1 mm wide), typically micropolydesmid facies (small paraterga, large and clubbed tergal setae arranged in three transverse rows etc.), strongly enlarged gonocoxae that fully conceal the telopodites and, above all, male legs 2, especially their tibiae, greatly enlarged compared to others (Cook 1896b). No number of body segments has been given.

### Dendrobrachypus

Taxon classificationAnimaliaPolydesmidaTrichopolydesmidae

Verhoeff, 1941

#### Type-species.

*Dendrobrachypuspusillus* Verhoeff, 1941, by monotypy; Fernando Po.

Twenty segments (male, female), body length 5–5.5 mm. Male head without epicranial modifications. Paraterga moderate, tergal setae medium-sized and bacilliform. Gonopodal coxa very large, concealing most of telopodite; the latter’s apical part with only one branch (ab); seminal groove simple and rather long, ending subapically on a long solenomere (sl) (Figure 2D).

### Eburodesmus

Taxon classificationAnimaliaPolydesmidaTrichopolydesmidae

Schubart, 1955

#### Type-species.

*Eburodesmuserectus* Schubart, 1955, by original designation; Guinea, Côte d’Ivoire.

Twenty segments (male, female); body length; 5.5–6 mm. Male head without epicranial modifications. Paraterga modest, tergal setae medium-sized and bacilliform. Gonopodal coxae very large; gonocoel very deep, each coxa apically with an evident process (cp) directed mesally; seminal groove forming a very clear loop before running onto a long and simple solenomere (sl); apical branch (ab) of telopodite forming a strong finger directed basad; middle part with a long straight process (bb) directed apicad (Figure 2E).

### Elgonicola

Taxon classificationAnimaliaPolydesmidaTrichopolydesmidae

Attems, 1939

#### Type-species.

*Elgonicolajeanneli* Attems, 1939, by original designation; Uganda.

Twenty segments (male, female); body length 8 mm. Male head without epicranial modifications. Paraterga moderate, tergal setae very long, slender and bacilliform. Gonopodal coxae very large, gonocoel deep, concealing most of telopodite; seminal groove short and straight, ending subapically on a short solenomere (sl); apical part of telopodite with two small branches (ab and bb), one of which (ab) a slender hook (Figure 1D).

### Hemisphaeroparia

Taxon classificationAnimaliaPolydesmidaTrichopolydesmidae

Schubart, 1955

#### Type-species.

*Hemisphaeropariacumbula* Schubart, 1955, by original designation; Guinea, Côte d’Ivoire.

Twenty segments (male, female); body length 3.5–4 mm. Male head without epicranial modifications. Paraterga moderate, tergal setae medium-sized and bacilliform. Gonopodal coxae very large, gonocoel deep, concealing most of telopodite; seminal groove short, ending on a simple finger-shaped solenomere (sl); telopodite basically tripartite, with a large, central, sac-shaped part and two slightly higher, subflagelliform, adjacent, lateral branches (ab and bb) (Figure 2F, G).

### Heterosphaeroparia

Taxon classificationAnimaliaPolydesmidaTrichopolydesmidae

Schubart, 1955

#### Type-species.

*Heterosphaeropariavilliersi* Schubart, 1955, by original designation; Guinea, Côte d’Ivoire.

Twenty segments (male, female); body length 5–6 mm. Male head without epicranial modifications. Paraterga relatively well developed, tergal setae long and bacilliform. Gonopodal coxae large, each with an evident apical process (cp) laterally, gonocoel shallow, leaving most of telopodite exposed; seminal groove rather short and straight, ending on a simple, long, finger-shaped solenomere (sl); telopodite basically bipartite with two subequal, high, ribbon-shaped processes/branches (ab and bb) distal to solenomere (Figure 2A).

### Mabocus

Taxon classificationAnimaliaPolydesmidaTrichopolydesmidae

Chamberlin, 1951

#### Type-species.

*Mabocusgranulifer* Chamberlin, 1951, by original designation; Angola.

Twenty segments (male, female); body width 0.6 mm. Male head with a central, boletiform, epicranial projection. Paraterga relatively well developed, tergal setae medium-sized, bacilliform to slightly clavate. Male coxae moderately large, gonocoel relatively small; telopodite well exposed, distal part with a single apical branch (ab) subdivided into two on top. Both seminal groove and solenomere (sl) short and simple, the latter spiniform (Figure 1E, F). Poorly described originally (Chamberlin 1951), properly redescribed from the holotype by Kraus (1958).

### Mecistoparia

Taxon classificationAnimaliaPolydesmidaTrichopolydesmidae

Brolemann, 1926

#### Type-species.

*Mecistoparialophotocrania* Brolemann, 1926, by original designation; Dahomey.

Nineteen segments (male, female); body length 3–3.5 mm. Male head without epicranial modifications. Paraterga moderate; setae long and bacilliform. Gonopodal coxae very strongly developed; gonocoel very deep, almost fully concealing telopodite; seminal groove very short and simple, ending on a long simple solenomere (sl); telopodite with a long apical finger-shaped branch (ab) directed basaly and a shorter finger-shaped branch (bb) parabasally; telopodite bipartite (Figure 2B, C).

### Megaloparia

Taxon classificationAnimaliaPolydesmidaTrichopolydesmidae

Brolemann, 1920

#### Type-species.

Sphaeroparia (Megaloparia) lignivora Brolemann, 1920, by subsequent designation by Attems (1940); Kenya.

Twenty segments (male, female); body length 6.5 mm. Male head without epicranial modifications. Paraterga moderate, tergal setae very short. Gonopodal coxae unusually small, leaving telopodite mostly exposed and suberect; telopodite bipartite, seminal groove short and simple, ending on a short solenomere (sl) with a hairy pulvillus squeezed between both branches ab and bb (Figure 1B).

### Physetoparia

Taxon classificationAnimaliaPolydesmidaTrichopolydesmidae

Brolemann, 1920

#### Type-species.

Sphaeroparia (Physetoparia) imbecilla Brolemann, 1920, by monotypy; Kenya.

Body length 7.5 mm; paraterga moderate. Male head without epicranial modifications. Gonopodal coxae very large; gonocoel deep, containing most of telopodite; both solenomere (sl) and seminal groove short, apical branch (ab) very large (Figure 1C).

### Sphaeroparia

Taxon classificationAnimaliaPolydesmidaTrichopolydesmidae

Attems, 1909

#### Type-species.

*Sphaeropariaminuta* Attems, 1909, by monotypy; Tanzania.

Nineteen segments (male, female); body length 6–6.5 mm. Genae of male much larger than those of female. Male head without epicranial modifications. Paraterga moderate; setae medium-sized and bacilliform. Gonopodal telopodite tripartite; both coxae and gonocoel relatively small; telopodite well-exposed; distalmost branch (ab) particularly large and high, with an apical tooth; seminal groove relatively short and simple, ending on a long simple solenomere (sl); parabasal (= prefemoral) part as usual, clearly setose; middle part a large scapuliform lobe (bb) with a long and thin flagellum (fl) near base (Figure 1A).

### Trichozonus

Taxon classificationAnimaliaPolydesmidaTrichopolydesmidae

Carl, 1905

#### Type-species.

*Trichozonusescalerae* Carl, 1905, by monotypy; Equatorial Guinea.

#### Female.

20 segments, body length 8 mm; paraterga modest, tergal setae long and bacilliform. Like *Bactrodesmus*, this genus remains too poorly documented to consider in our following analysis.

The above brief accounts are given to reiterate the foundations of the previous classification and to offer a new one below. The classification developed by Mauriès and Heymer (1996) in their review of the African Trichopolydesmidae (= Fuhrmannodesmidae) cannot be accepted because it totally lacks any evolutionary perspective. These authors themselves admit their approach as being “bastard” and overly lumping, as they placed all species but one (*Bactrodesmus*, dubious) in the single genus *Sphaeroparia*, within which they accepted six subgenera and suggested several synonymies.

Golovatch (1992, 1994) provided an evolutionary scenario for the genera of Trichopolydesmidae (= Fuhrmannodesmidae) known from South America, accepting as the basalmost those genera showing rather small, narrowly fused^1^, subglobose gonopodal coxae that form no significant gonocoel in which to hinge the largely exposed, usually rather complex and elongate telopodites. Amongst the Afrotropical trichopolydesmids such are the genera *Elgonicola*, *Heterosphaeroparia*, *Megaloparia*, and *Sphaeroparia* (Figs 1D, 2A, 1B, and 1A, respectively). At the opposite end which obviously represents the evolutionary summit are such genera as *Dendrobrachypus*, *Eburodesmus*, and *Mecistoparia* (Figs 2D, E and 2B-C, respectively). Their gonopodal coxae are especially voluminous and inflated laterad; the particularly deep gonocoel is capable of concealing entire or nearly entire telopodites. Sometimes the coxa shows a rather conspicuous apicolateral process or lobe, this being suberect (*Heterosphaeroparia*, Figure 2A, cp) or directed more or less mesally (*Eburodesmus*, Figure 2E, cp). Schubart (1955) created his *Eburodesmus*, based on such evident and more or less mesally directed coxal processes (Figure 2E, cp), but we think this may be only a species-level character occasionally present also in distinctly more basal taxa, e.g., *Heterosphaeroparia* (Figure 2A, cp). A series of transitions can be seen between the two extremes, e.g., in *Heterosphaeroparia*, *Elgonicola* or *Hemisphaeroparia*, when 1–3 prominent branches project well beyond the coxal margin, while the telopodite is mainly deeply sunken inside the coxa. Typically there are 2+2 particularly strong setae near the place of central fusion of both coxae, while their lateral surfaces are normally more or less clearly granulate/alveolate and setose.

The cannula in Afrotropical Trichopolydesmidae is invariably medium-sized, tube-shaped, strongly curved, long, and slender, its tip entering the densely setose, funnel-shaped, “prefemoral” part of the telopodite which extends from base to apex. The seminal groove is mostly rather short and straight, usually running on the mesal side to end on a simple, more or less finger- or spine-shaped, sometimes retrorse solenomere (sl). In a few cases, the solenomere ends near a kind of hairy pulvillus or shows a tooth at its base. However, there is a remarkable exception, when the seminal groove makes a distinct loop before proceeding onto a prominent solenomere (e.g., *Eburodesmus*, Figure 2E). Such situations seem to imply telopodite torsion. The length of a solenomere is usually barely more than species-specific, but the course of the seminal groove is definitely a generic-level character, as can be seen in Neotropical or Southeast Asian Trichopolydesmidae (Golovatch 1992, 1994, Golovatch et al. 2014, Golovatch and VandenSpiegel 2016).

The gonopodal telopodite is typically helmet- or boat-shaped. At the apicolateral end which is opposite to the “prefemoral” funnel there is a group of partly especially stiff setae usually extending basad across or over most of the funnel. Variations in the shape of the remaining parts of the telopodite are especially prominent. Among the most common outgrowths or processes of the telopodite we choose to denominate the following. A unipartite telopodite which shows only a single prominent branch, apical in position (ab), is observed in *Dendrobrachypus*, *Mabocus*, *Mecistoparia* and *Physetoparia* (Figs 2D, 1E-F, 2B–C and 1C, respectively). Bi- or tripartite telopodites are more usual, sometimes also supplied with evident central lobes (lo). The branch which is the closest to the apex is denominated an apical branch (ab), while that lying the closest to the basal part is termed a basal branch (bb). If there is another branch located between ab and bb, this is a medial branch (mb). Two of or all three branches are often strongly adjacent to each other. A few species groups may be provisionally outlined based on the general conformation of the gonopodal telopodites.

Two different general trends can be observed in the evolution of the gonopods in Afrotropical Trichopolydesmidae. While the coxa tends to grow and develops an increasingly prominent gonocoel, the originally relatively complex and largely exposed telopodite, at least in some cases, seems to shrink gradually and often becomes simplified. Indeed, the presumably basal taxa with particularly small gonocoxae (e.g., *Sphaeroparia*, *Elgonicola* or *Megaloparia*, Figs 1A, 1D and 1B, respectively) show complex apical structures on telopodites. In cases like *Eburodesmus* (Figure 2E) and *Heterosphaeroparia* (Figure 2), it is the coxal apicolateral process (cp) that takes the protective function to secure the still complex and exposed parts of the telopodite. Usually, when the outgrowths are mostly concealed and little exposed beyond the coxa, they are particularly few and sometimes reduced just to one branch (e.g., *Dendrobrachypus*, Figure 2D or *Mecistoparia*, Figure 2B, C). Interestingly, the single species of *Sphaeroparia*, *S.simplex* Golovatch, 2013, described from the Balkans and several Greek islands (Golovatch 2013), i.e., well beyond the Afrotropical realm, shows voluminous gonopodal coxae and especially simple and small telopodites, these latter being mostly concealed inside the gonocoel. Now that *Sphaeroparia* receives a clear definition (see below) and joins the basal group of genera with still small gonocoxae and complex and well-exposed telopodites, the identity and generic allocation of *S.simplex* must be revised (Vagalinski et al., in preparation).

When a delicate solenomere is left well-exposed (Figs 1C, 2D, F-G), it appears to always be protected by adjacent stronger structures serving as a solenophore. The number and shapes of such accessory outgrowths (processes and lobes) varies between species, but they are always few (1–3) and tend to be little exposed to fully concealed or even absent in more advanced genera.

Such are the main guidelines, all based solely on gonopodal anatomy, to follow in order to obtain new generic delimitations arranged according to an increasing complexity of the coxae, combined with a decreasing complexity of the telopodites. Somatic characters such as the number of body segments (19 in the male or in both sexes, or 20 in both sexes), the pore formula (always normal, but can be a little abbreviated on the last 1–2 segments before the telson: 5, 7, 9, 10, 12, 13, 15–17(18,19)), the degree of development of paraterga (usually moderate, often small, but never really well-developed and strongly flattened dorsally), the position of the ozopores (usually open flush dorsally near the caudal corner of pore-bearing paraterga), the shape of tergal setae (short and clavate to very long and bacilliform), the presence of modifications on male legs 2 (enlarged in *Bactrodesmus*) and on the male head (ranging sporadically from nothing to a strong, central bulge or a mushroom-like or bulbous tubercle), they all are considered here as species-specific. This situation agrees with general wisdom derived from other tropical faunas (Golovatch 1994, Golovatch et al. 2014).

A somatic character that deserves special mention is the unusually strongly developed peri-spiracular structure on segment 2. In all new species described below, the spiracle is located on a high finger with a complex tip placed next to coxa 2 (Figs 11K, 21J-L). Is this a generic feature that characterizes *Hemisphaeroparia*? Could Cook (1896b) have mistaken the spiracle for leg 2 in his *Bactrodesmus*?

The generic reclassification presented below considers only the type species, leaving aside the other component species and their allocations to our next paper on Afrotropical Trichopolydesmidae (Golovatch et al., in preparation). Two nominal genera, *Bactrodesmus* and *Trichozonus*, are dubious and are for the time being to be discarded from further consideration, because their gonopodal characters remain unknown. Whereas *B.claviger* is generally possible to revise or recognize based either on type or topotypic material from Liberia, since male legs 2 of this species are said to be conspicuously enlarged (Cook 1896b), the identity of *T.escalerae* is bound to remain obscure (Carl 1905) until a male topotypic sample from Fernando Po becomes available for study.

### Genus Sphaeroparia

Taxon classificationAnimaliaPolydesmidaTrichopolydesmidae

Attems, 1909

#### Type-species:

*Sphaeropariaminuta* Attems, 1909

#### Synonym:

*Megaloparia* Brolemann, 1920, synonymized by Mauriès and Heymer (1996: 167) (type-species: *Sphaeroparialignivora* Brolemann, 1920)

#### Diagnosis.

Both gonopodal coxae and gonocoel relatively small; telopodite strongly exposed, complex, with at least two strong branches (ab and bb, the latter may be subdivided into two, including fl); seminal groove short and simple, solenomere on caudal face, shorter or longer, spiniform, with or without a pulvillus (pu) near end (Figure 1A).

#### Remark.

This genus is presumably among the basalmost representatives of Afrotropical Trichopolydesmidae.

### Genus Physetoparia

Taxon classificationAnimaliaPolydesmidaTrichopolydesmidae

Brolemann, 1920 stat. n.

#### Type-species.

*Sphaeropariaimbecilla* Brolemann, 1920

#### Synonyms.

*Elgonicola* Attems, 1939, syn. n. (type-species: *Elgonicolajeanneli* Attems, 1939); *Mabocus* Chamberlin, 1951, syn. n. (type-species: *Mabocusgranulifer* Chamberlin, 1951); *Heterosphaeroparia* Schubart, 1955, syn. n. (type-species: *Heterosphaeropariavilliersi* Schubart, 1955)

#### Diagnosis.

Both gonopodal coxae and gonocoel medium-sized; telopodite usually less strongly exposed and less complex (when strongly exposed, then with a protective coxal apicolateral process), with 1–2 strong branches (ab or ab and bb); seminal groove short and simple, solenomere shorter or longer, sometimes exposed, subspiniform (Figs 1C, D, E–F, 2A).

#### Remarks.

This genus is among the more advanced representatives of Afrotropical Trichopolydesmidae. Mauriès and Heymer (1996: 168) regarded it as a subgenus of *Sphaeroparia*, with *Hemisphaeroparia* and possibly also *Dendrobrachypus* and *Trichozonus* as synonyms of *Physetoparia*.

### Genus Mecistoparia

Taxon classificationAnimaliaPolydesmidaTrichopolydesmidae

Brolemann, 1926

#### Type-species.

*Mecistoparialophotocrania* Brolemann, 1926

#### Synonym.

*Dendrobrachypus* Verhoeff, 1941, syn. n. (Type-species: *Dendrobrachypuspusillus* Verhoeff, 1941

#### Diagnosis.

Both gonopodal coxae and gonocoel medium-sized; telopodite usually less strongly exposed and complex (when strongly exposed, then with a protective coxal apicolateral process), with 1–2 strong branches (ab or ab and bb); seminal groove short and simple, solenomere shorter or longer, sometimes exposed, subspiniform (Figs 1B, 2D).

#### Remarks.

This genus is among the more advanced representatives of Afrotropical Trichopolydesmidae. Mauriès and Heymer (1996: 168) regarded it as a subgenus of *Sphaeroparia*.

### Genus Eburodesmus

Taxon classificationAnimaliaPolydesmidaTrichopolydesmidae

Schubart, 1955, stat. revalid.

#### Type-species.

*Eburodesmuserectus* Schubart, 1955

#### Diagnosis.

Both gonopodal coxae and gonocoel very large; telopodite only barely exposed, but complex (this possibly being in correlation that each coxa has a protective apicolateral process), with two strong branches (ab and bb); seminal groove long and forming a conspicuous loop before moving onto a caudally located solenomere (Figure 2E).

#### Remarks.

This genus is among the most advanced representatives of Afrotropical Trichopolydesmidae. Mauriès and Heymer (1996: 168) regarded it as a subgenus of *Sphaeroparia*.

### Genus Hemisphaeroparia

Taxon classificationAnimaliaPolydesmidaTrichopolydesmidae

Schubart, 1955, stat. revalid.

#### Type-species.

*Hemisphaeropariacumbula* Schubart, 1955

#### Diagnosis.

Both gonopodal coxae and gonocoel large to very large; telopodite usually moderately to barely exposed, but complex, with 1–3 strong branches (ab and/or bb, or ab, mb and bb, occasionally also with a lobe more basally), only sometimes with a single particularly strongly exposed branch (ab); seminal groove mostly short, solenomere only sometimes absent, but usually finger-shaped and located caudomesally (Figure 2F, G).

#### Remarks.

This genus is among the most advanced representatives of Afrotropical Trichopolydesmidae. Mauriès and Heymer (1996: 168) regarded it as a synonym of *Physetoparia*. All trichopolydesmid species treated below from Cameroon appear to belong to this genus, albeit forming a few species groups.

Based on the numerous new and one old species from Cameroon treated below, the diagnosis of *Hemisphaeroparia* can be updated as follows.

#### Updated diagnosis.

Body with 19 or 20 segments. Male epicranial modifications present or absent. Spiracle next to coxa 2 conspicuously enlarged, finger- or mushroom-shaped and with a complex tip. Both gonopodal coxae and gonocoel large to very large; telopodite usually moderately to barely exposed, but complex, with 1–3 strong branches (ab and/or bb, or ab, mb and bb, occasionally also with a lobe more basally), only sometimes with a single particularly strongly (ab) or considerably (bb) exposed branch; seminal groove mostly short, solenomere only sometimes absent, but usually transverse (= directed anteriorly), finger-shaped or spiniform, and located caudomesally.

## Species descriptions

### Hemisphaeroparia zamakoe sp. n.

Taxon classificationAnimaliaPolydesmidaTrichopolydesmidae

http://zoobank.org/1D9169D7-FF64-46F0-92BC-D69AED8802CF

Figs 3 , 4 , 28A

#### Type material.

Holotype ♂ (MRAC 22735), Cameroon, Center Region, Zamakoe Forest, 03°33’N, 011°31’E, 815 m a.s.l., forest, 20.IX.2014, leg. A.R. Nzoko Fiemapong and C. Oumarou Ngoute.

#### Paratypes.

6 ♂♂ (MRAC 22736), 1 ♂ (SEM, MRAC 22737), same locality, 18.IV.2015; 1 ♂ (without gonopods, either lost or mounted on slide) (MRAC 22738), 1 ♂ (ZMUM), same locality, 19.IV.2014; 1 ♂ (ARNF), same locality, 21.III.2015, all leg. A.R. Nzoko Fiemapong and C. Oumarou Ngoute.

#### Diagnosis.

Differs from all other species of the genus by the presence of a boletiform epicranial tubercle (♂), coupled with unusually densely setose gonopodal telopodites which are deeply sunken inside a large gonocoel and show only two, slender, contiguous, little-exposed branches (ab, bb), both followed by a small, round, fully concealed lobe (lo) more basally (Figure 4).

#### Name.

To emphasize the type locality; noun in apposition.

#### Description.

Length of holotype ca. 5 mm (♂), width of midbody pro- and metazonae 0.4 and 0.6 mm (♂), respectively. Length of paratypes 5–7 mm, width of midbody pro- and metazonae 0.4–0.5 and 0.6–0.7 mm (♂), respectively. Coloration in alcohol uniformly pallid (Figure 28A).

**Figure 3.**
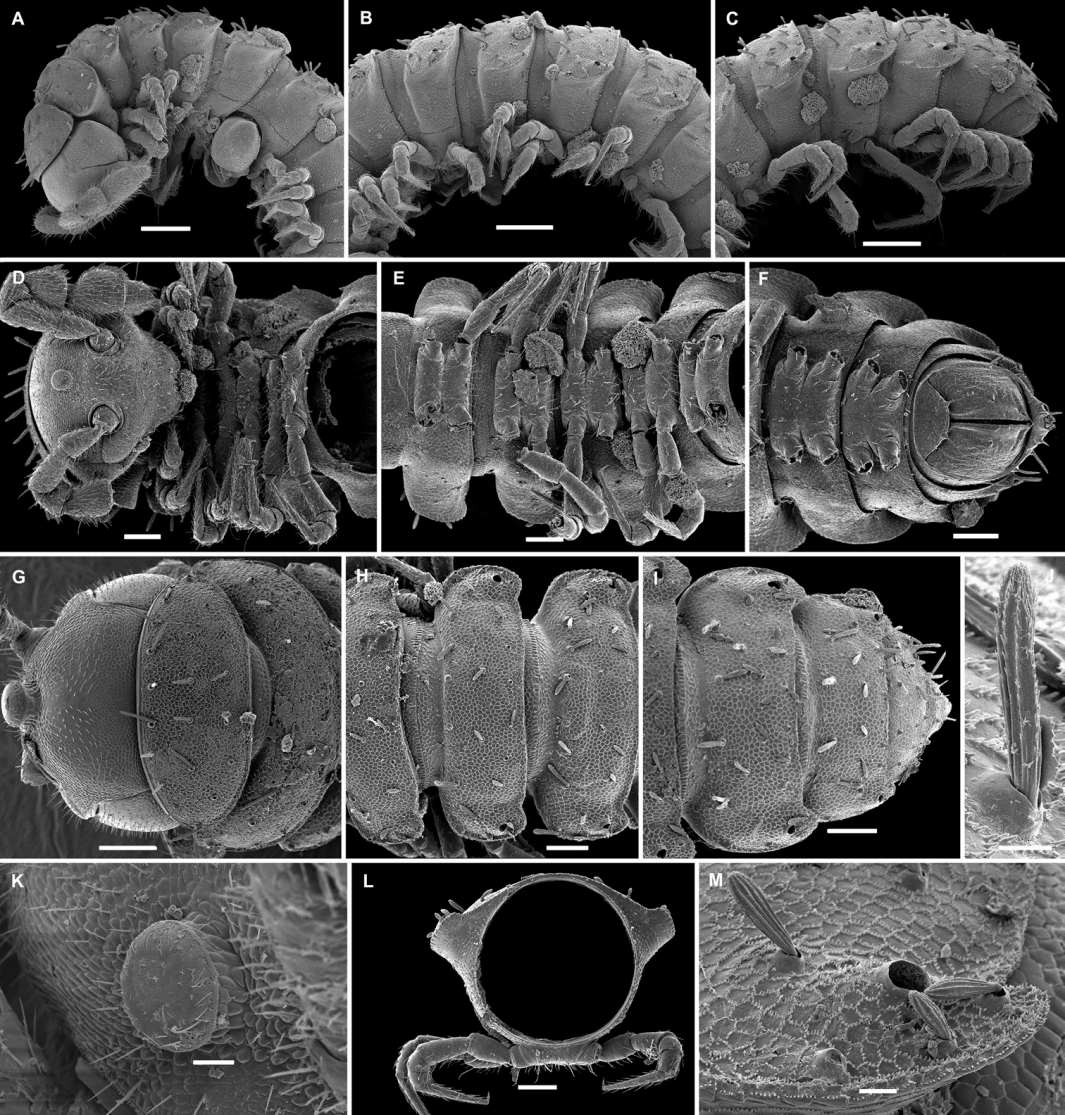
*Hemisphaeropariazamakoe* sp. n., SEM micrographs of ♂ paratype **A, D, G** anterior part of body, lateral, ventral and dorsal views, respectively **B, E, H** midbody segments, lateral, ventral and dorsal views, respectively **C, F, I** posterior part of body, lateral, ventral and dorsal views, respectively **J** tergal seta, lateral view **K** epicranial tubercle, subdorsal view **L** midbody segment, caudal view **M** midbody paratergum, lateral view. Scale bars: 0.1 mm (**A–I, L**), 0.05 mm (**K, M**), 0.01 mm (**J**).

Body with 20 segments. Tegument very delicately micro-alveolate, mainly slightly shining. Head very densely micropilose, with a very distinct, mushroom-like, frontal tubercle (♂) (Figure 3D, G, K). Interantennal isthmus almost two times as large as diameter of antennal socket. Antennae long and strongly clavate, reaching back to segment 2 or 3 when stretched dorsally (♂). In length, antennomere 3 = 6 > 2 = 5 > 1 = 4 =7; antennomere 6 the largest, antennomeres 5 and 4 each with a distinct, round, distodorsal field of sensilla. In width, collum < head < segments 2–4 < 5–16; thereafter body gradually tapering towards telson. Collum ellipsoid, transversely oval, like all following metaterga with three transverse, regular rows of setae. Tergal setae medium-sized, each ca. 1/5 as long as metatergum, bacilliform and longitudinally ribbed (Figure 3A-C, G-J, L, M), always 3+3 in each row on postcollum metaterga. An extremely faint transverse sulcus visible behind first row on some metaterga. Dorsum invariably regularly convex. Paraterga medium-sized, set at around upper 1/3 of metazonae (Figure 3A-C, L), visible starting with collum, often slightly upturned caudally, faintly, but regularly rounded and bordered, lateral incisions absent. Caudal corner of paraterga always rounded, drawn increasingly back, but reaching beyond rear tergal margin only on segments 17 and 18 (Figure 3I). Pore formula normal, only slightly abbreviated: 5, 7, 9, 10, 12, 13, 15–18. Ozopores small, round, opening flush dorsally near caudal corner of poriferous paraterga. Stricture between pro- and metazonae wide, shallow. Limbus very finely microspiculate. Segment 2 with an unusually prominent, tuberculiform and apically complex spiracle on each side. Pleurosternal carinae traceable as very faint lines on most segments (Figure 3A–C). Epiproct short, conical, flattened dorsoventrally. Hypoproct semi-circular, setae strongly separated and borne on minute knobs.

Sterna wide, unmodified, setose. Legs rather long and slender, ca. 1.1–1.2 times as long as midbody height; in length, tarsus > femur > coxa = prefemur = postfemur = tibia, the latter with a particularly long, tactile seta apicodorsally. Tarsal brushes present (♂).

**Figure 4.**
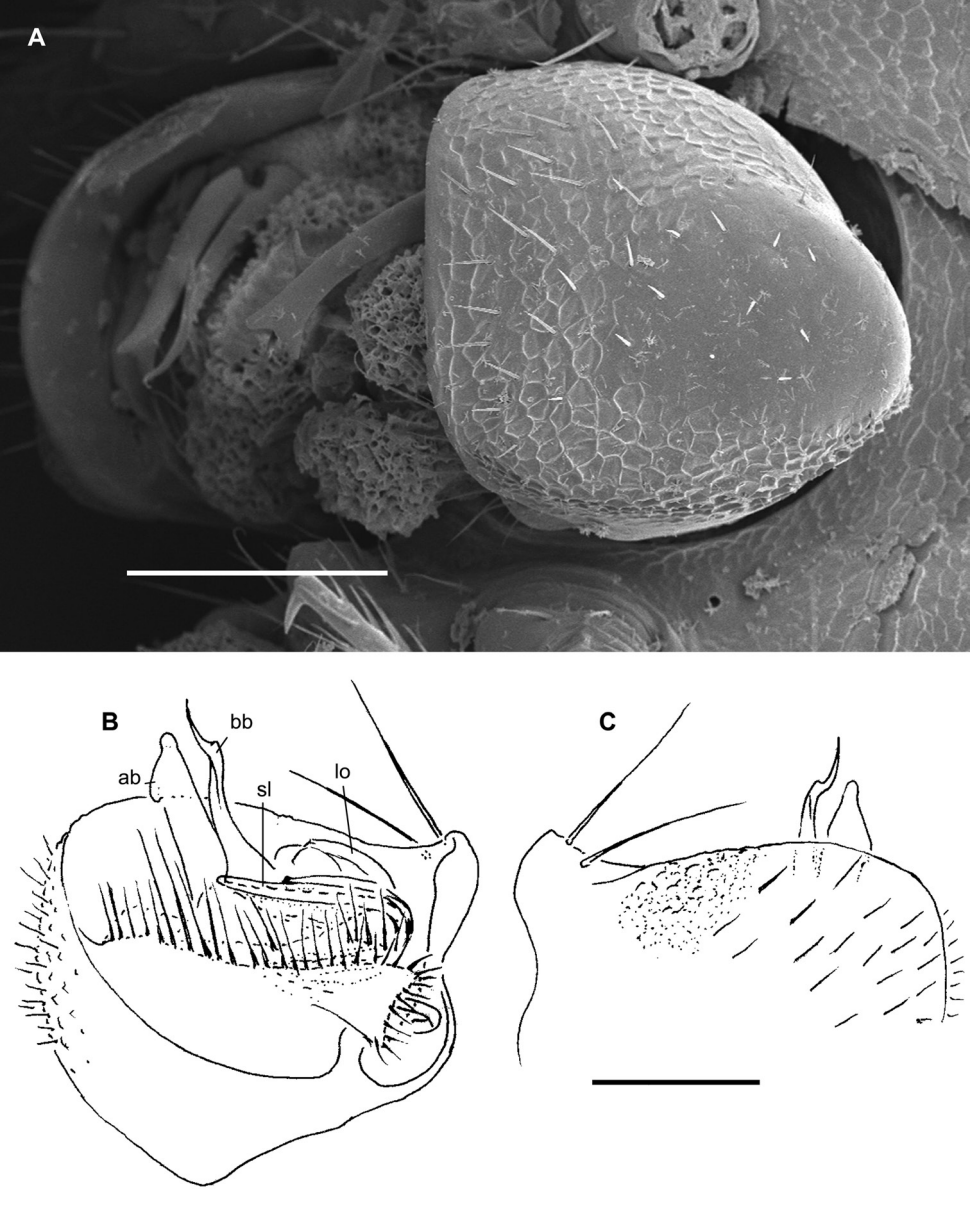
*Hemisphaeropariazamakoe* sp. n., ♂ paratypes **A**SEM micrograph of both gonopods in situ, ventrolateral view **B, C** right gonopod, mesal and lateral views, respectively. Scale bars: 0.1 mm. Abbreviations: **ab** apical branch of telopodite, **bb** basal branch of telopodite, **lo** lobe, **sl** solenomere.

Gonopods (Figure 4) with large, subglobose, clearly exposed, alveolate coxae, these rather densely setose nearly throughout, fused medially at base, each carrying two very long setae near place of fusion. Telopodites almost fully concealed inside a very large gonocoel, each very densely setose, with only two branches (ab, bb), both contiguous and only slightly exposed beyond coxa, followed by a small round lobe (lo) more basally. Seminal groove short, moving onto a long, subspiniform solenomere (sl), in mesal view the latter subtransverse and directed apicolaterad.

### Hemisphaeroparia bangoulap sp. n.

Taxon classificationAnimaliaPolydesmidaTrichopolydesmidae

http://zoobank.org/CA5FE0D5-B0A6-4C48-9443-C51A3B69B946

Figs 5 , 6 , 28B

#### Type material.

Holotype ♂ (MRAC 22739), Cameroon, West Region, Bangoulap, sacred forest (slightly disturbed), 6°00’N, 10°34’E, 13.X.2017, leg. A.R. Nzoko Fiemapong.

Paratypes. 2 ♂ (both without gonopods, either lost or mounted on slide)(MRAC 22740), 1 ♂ (SEM, MRAC 22741), same locality, together with holotype.

#### Diagnosis.

Differs from other species of the genus by the presence of a boletiform epicranial tubercle (♂), coupled with well-exposed gonopodal telopodites that show not only slender branches ab and bb, but each also a well-expressed lobe (lo) with an unusually deep transverse gutter (g) with very strongly thickened walls at the base, as well as a vestigial solenomere (sl) with a remarkable process (t) near its base (Figure 6).

**Figure 5.**
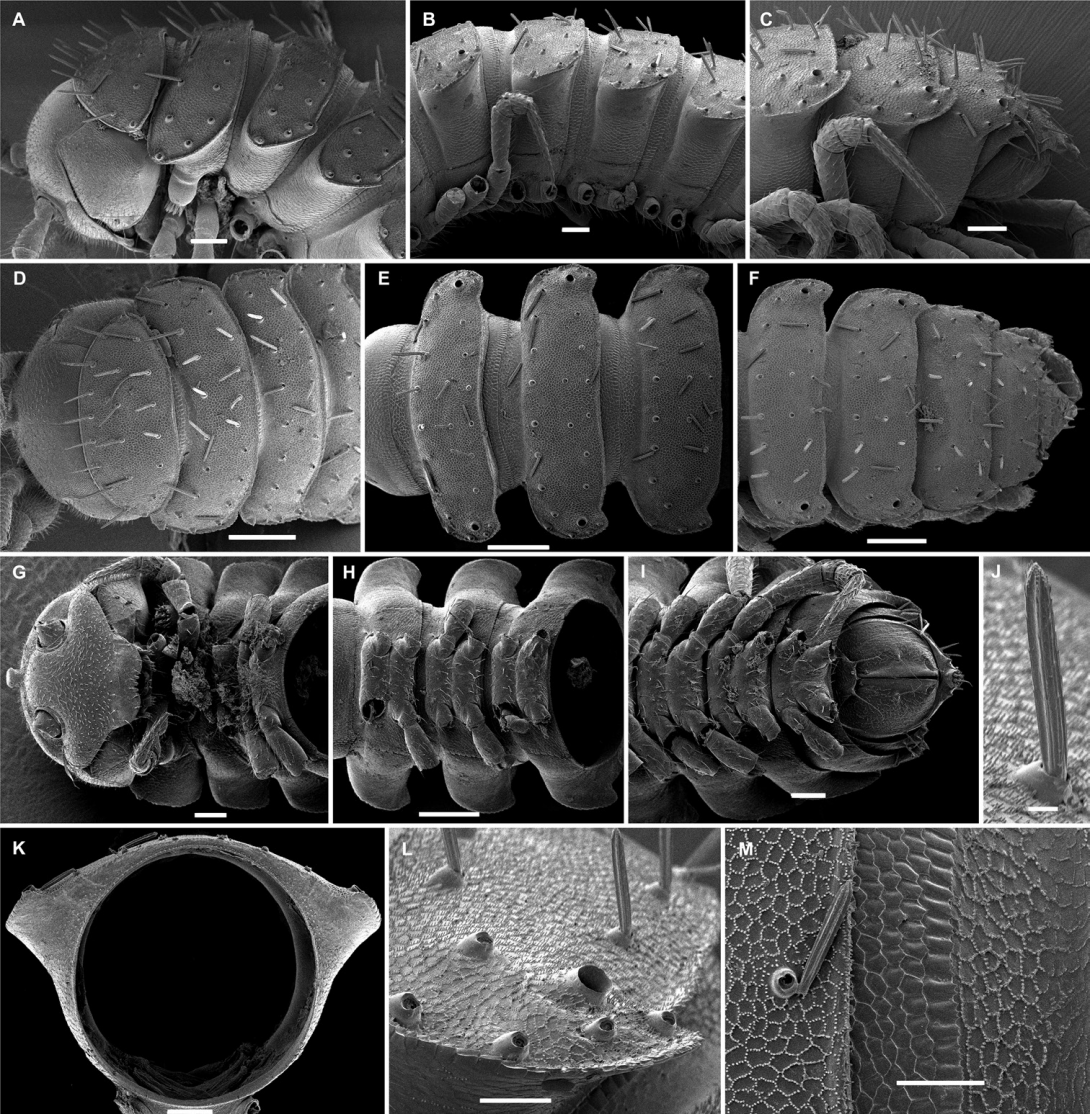
*Hemisphaeropariabangoulap* sp. n., SEM micrographs of ♂ paratype **A, D, G** anterior part of body, lateral, dorsal and ventral views, respectively **B, E, H** midbody segments, lateral, dorsal and ventral views, respectively **C, F, I** posterior part of body, lateral, dorsal and ventral views, respectively **J** tergal seta, lateral view **K** midbody segment, caudal view **L** midbody paratergum, lateral view **M** tergal fine structure. Scale bars: 0.2 mm (**D–F, H**), 0.1 mm (**A–C, G, I, K**), 0.05 (**L, M**), 0.01 mm (**J**).

#### Name.

To emphasize the type locality; noun in apposition.

#### Description.

Length of holotype ca. 6 mm (♂), width of midbody pro- and metazonae 0.5 and 0.7mm (♂), respectively. Length of paratypes 5.5–6 mm, width of midbody pro- and metazonae 0.5 and 0.7–0.75 mm (♂), respectively. Coloration in alcohol light pinkish to light pinkish brown, metaterga and antennae marbled red-brown. Prozonae, venter, and legs light yellowish grey (Figure 28B).

All other characters as in *H.zamakoe* sp. n., except as follows.

In width, collum < head < segment 3 < 3 = 4 < 5–16; thereafter, body gradually tapering towards telson.

**Figure 6.**
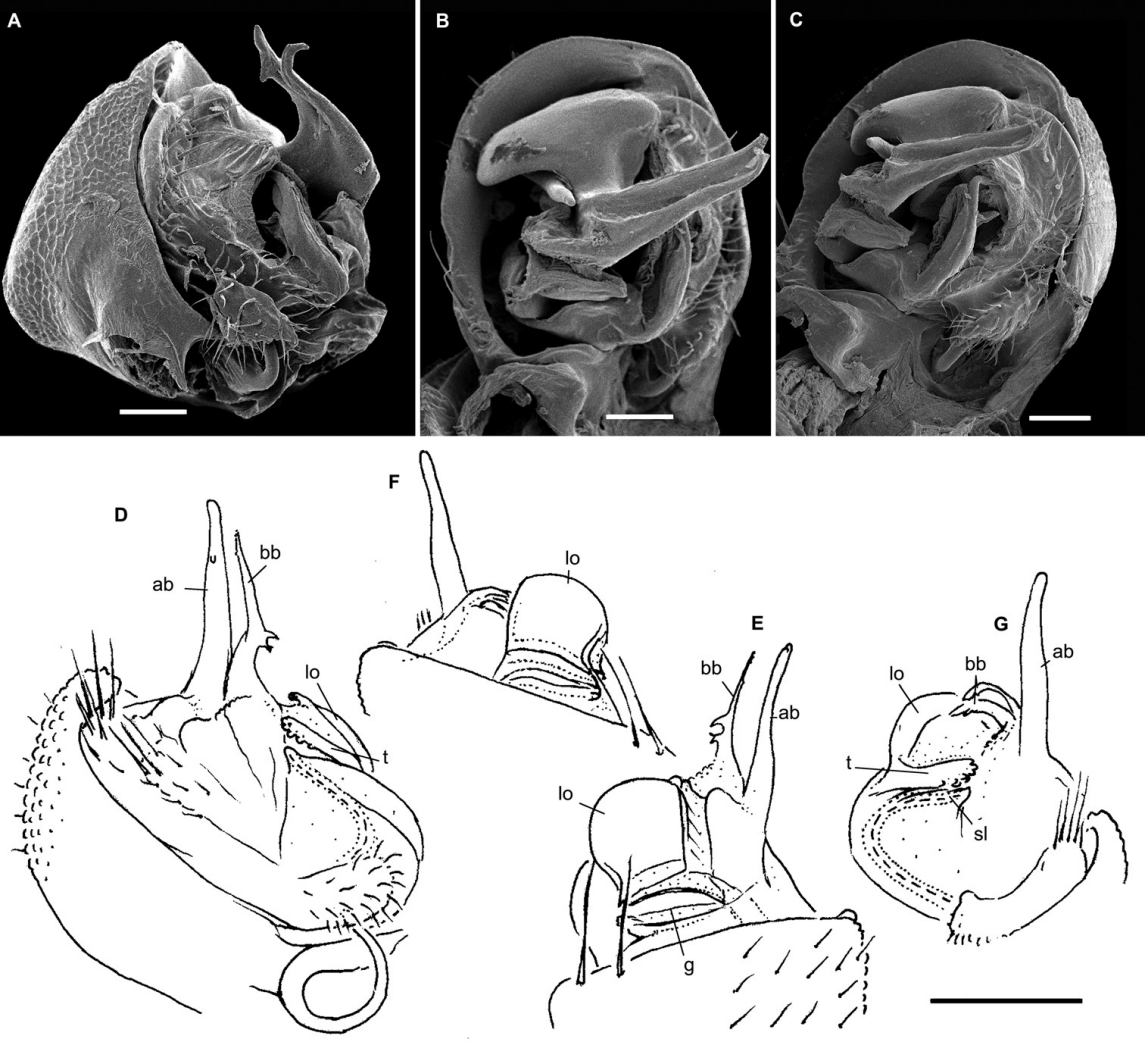
*Hemisphaeropariabangoulap* sp. n., ♂ paratypes **A–C**SEM micrographs of right gonopod, mesal, ventromesal, subventromesal views, respectively **D–G** right (**D, E**) and left (**F, G**) gonopods, mesal, lateral, mesal and lateral views, respectively. Scale bars: 0.05 (**A–C**), 0.2 mm (**D–G**). Abbreviations: **ab** apical branch of telopodite, **bb** basal branch of telopodite, **g** gutter, **lo** lobe, **sl** solenomere, **t** process.

Gonopodal telopodites more strongly exposed, less strongly setose, both main branches (ab, bb) longer, lobe (lo) also exposed, at base on lateral face with a prominent transverse gutter (g) with unusually strongly chitinized walls; branch bb sometimes subdivided into two flagelliform branchlets (Figure 6F, G). Seminal groove short, solenomere (sl) rudimentary, near its base with a conspicuous process (t).

### Hemisphaeroparia spiniger sp. n.

Taxon classificationAnimaliaPolydesmidaTrichopolydesmidae

http://zoobank.org/1AEABC29-FB50-4AB6-A625-2E81EAEBE3F3

Figs 7 , 8 , 28C

#### Type material.

Holotype ♂ (MRAC 22742), Cameroon, Center Region, Yaounde I University campus, palm plantation, 03°53’N, 011°30’E, 860 m a.s.l., 20.III.2018, leg. A.R. Nzoko Fiemapong.

Paratypes: 9 ♂♂ (MRAC 22743), 1 ♂ (without gonopods)(MRAC 22744), 4 ♀♀ (MRAC 22745). 1 ♂ (SEM, MRAC 22746), same locality, 18.IV.2015; 12 ♂♂ (MRAC: 22747), 3 ♂♂ (ZMUM), 3 ♂♂ (UY1), same locality, 7.IV.2014, all leg. A.R. Nzoko Fiemapong.

#### Diagnosis.

Differs from other species of the genus by the presence of a boletiform epicranial tubercle (♂), coupled with unusually densely setose gonopodal telopodites which are deeply sunken inside a large gonocoel and show not only two slender, little-exposed branches (ab, bb), followed by a small, round, fully concealed lobe (lo) more basally, but also a conspicuous transverse spine arising on the lateral side near the base of ab and bb (Figure 8).

#### Name.

To emphasize the long, transverse spine on the gonopodal telopodite; noun in apposition.

#### Description.

Length of holotype ca. 7 mm (♂), width of midbody pro- and metazonae 0.5 and 0.8 mm (♂), respectively. Length of paratypes 5.5–6 mm (♂, ♀), width of midbody pro- and metazonae 0.4–0.5 and 0.6–0.7 mm (♂) or 0.6 and 0.8 mm (♀), respectively. Coloration of holotype generally marbled red-brown, legs nearly pallid (Figure 28C). Paratypes mostly yellowish to nearly pallid.

All other characters as in *H.zamakoe* sp. n., except as follows.

**Figure 7.**
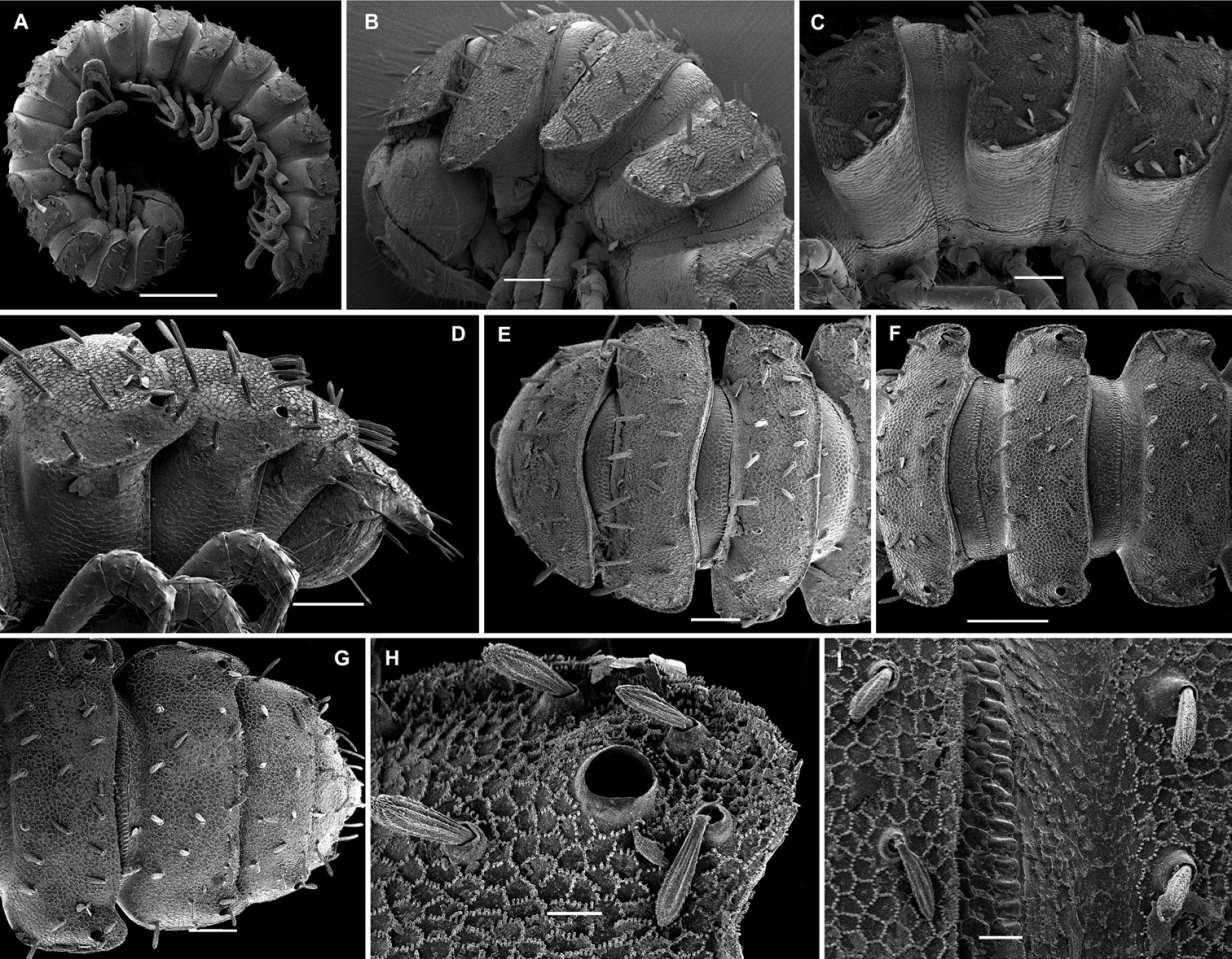
*Hemisphaeropariaspiniger* sp. n., SEM micrographs of ♂ paratype **A** habitus, lateral view **B, E** anterior part of body, lateral and dorsal views, respectively **C, F** midbody segments, lateral and dorsal views, respectively **D, G** posterior part of body, lateral and dorsal views, respectively **H** midbody paratergum, dorsolateral view **I** tergal fine structure. Scale bars: 0.5 mm (**A**), 0.1 mm (**B–G, J**), 0.02 mm (**H, I**).

Both ♂ and ♀ with 20 segments, but ♀ devoid of epicranial modifications. Antennae long and strongly clavate, reaching behind to segment 3 (♂) or 2 (♀) when stretched dorsally. In width, collum < head < segment 3 < 2 = 4 < 5–16; thereafter body gradually tapering towards telson. Tergal setae generally a little longer, ca. 1/3 to 1/4 as long as metatergum, bacilliform and ribbed (Figure 7A–C, G–J, L, M), arranged in two transvers rows on segments 2–7(8), thereafter in three rows (Figure 7A–G).

**Figure 8.**
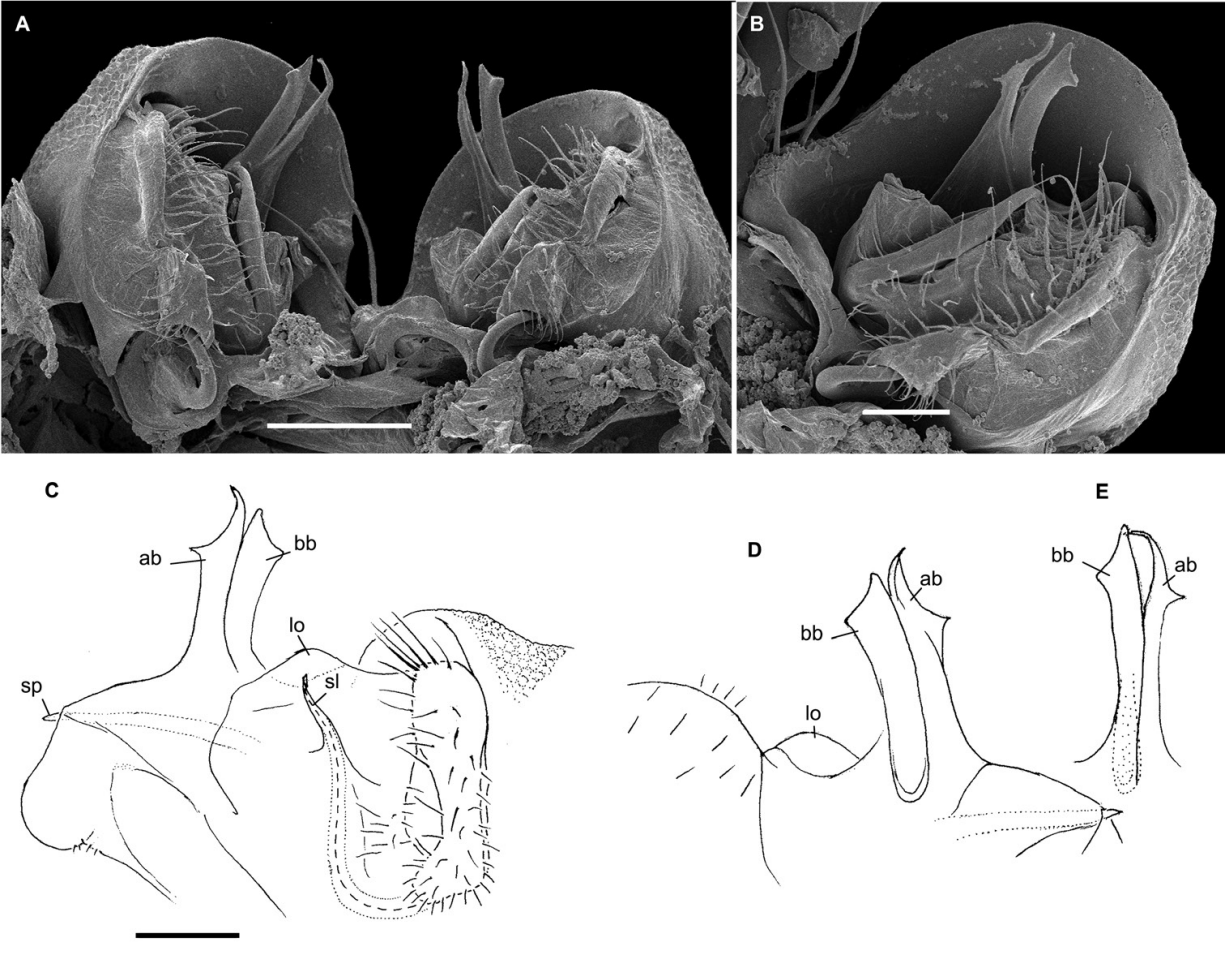
*Hemisphaeropariaspiniger* sp. n., ♂ paratypes **A**SEM micrographs of both gonopods in situ, caudal view **B**SEM micrograph of right gonopod, submesal view **C, D** right gonopod, mesal and lateral views, respectively **E** left gonod, branches ab and bb, mesal view. Scale bars: 0.1 mm (**A, C–E**), 0.5 mm (**B**). Abbreviations: **ab** apical branch of telopodite, **bb** basal branch of telopodite, **lo** lobe, **sl** solenomere, **sp** spiniform process.

Gonopods (Figure 8) forming a deep gonocoel, telopodites only slightly exposed through distal halves of their two main branches (ab, bb), both contiguous over most of their length and both subequal in shape and length, followed by a low rounded lobe (lo). Basal part of telopodite densely setose throughout; distobasal part with a conspicuous transverse spine (sp) arising near base of ab and bb on lateral side, but hidden on both sides. Seminal groove short, moving onto a longer or shorter solenomere (sl) on mesal side.

### Hemisphaeroparia ongot sp. n.

Taxon classificationAnimaliaPolydesmidaTrichopolydesmidae

http://zoobank.org/4AF63B91-FAEF-4D7B-8E93-E4A1A32CDE69

Figs 9 , 10 , 28D

#### Type material.

Holotype ♂ (MRAC 22748), Cameroon, Ongot Forest, 03°51’N, 011°25’E, ca. 810 m a.s.l., 26.III.2014, leg. A.R. Nzoko Fiemapong.

Paratypes: 1 ♂ (SEM, MRAC 22749), same locality, 27.IX.2014, leg. A.R. Nzoko Fiemapong; 1 ♀ (MRAC 22750), same locality, together with holotype.

#### Diagnosis.

Differs from all other species of the genus by the presence of a boletiform epicranial tubercle (♂), coupled with the caudal corner of paraterga becoming increasingly strongly drawn behind the rear tergal margin starting with segment 13 and the gonopodal telopodites that are deeply sunken inside a large gonocoel and show three main branches (ab, mb, bb), all well-exposed and followed by no lobe, as well as a short solenomere with a tooth (t) at base of both sl and ab (Figure 10).

#### Name.

To emphasize the type locality; noun in apposition.

#### Description.

Length of holotype ca. 5.5 mm (♂), width of midbody pro- and metazonae 0.45 and 0.6 mm (♂), respectively. Length of paratypes (♀) 5.5 mm, width of midbody pro- and metazonae 0.55–0.7 mm (♂), respectively. Coloration in alcohol nearly pallid to very light yellow (Figure 28D).

All other characters as in *H.zamakoe* sp. n., except as follows.

**Figure 9.**
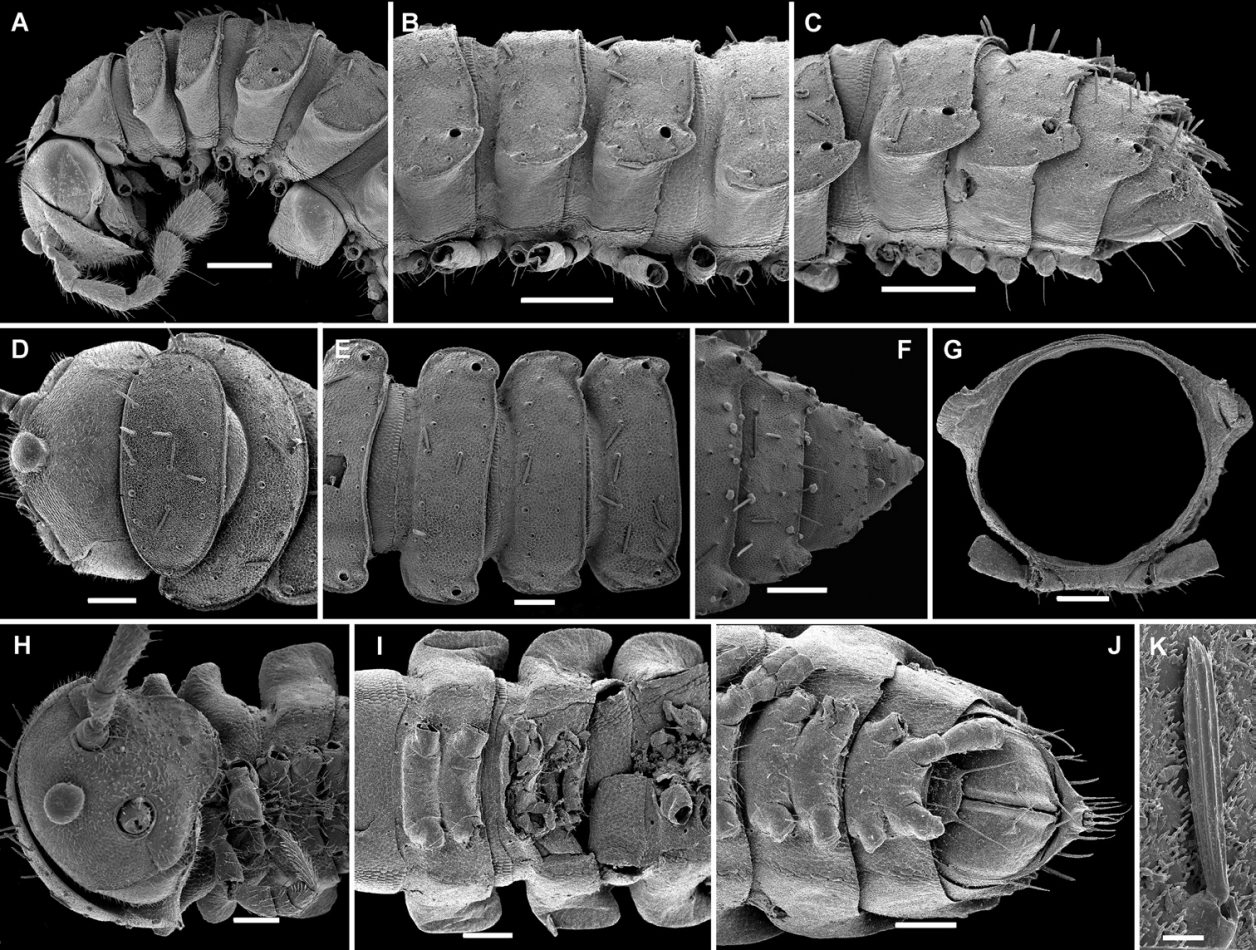
*Hemisphaeropariaongot* sp. n., SEM micrographs of ♂ paratype **A, D, H** anterior part of body, lateral, dorsal and ventral views, respectively **B, E, I** midbody segments, lateral, dorsal and ventral views, respectively **C, F, J** posterior part of body, lateral, dorsal and ventral views, respectively G midbody segment, caudal view **K** tergal seta, lateral view. Scale bars: 0.2 mm (**A–C**), 0.1 mm (**D–J)**, 0.01 mm (**K**).

Caudal corner of paraterga always rounded, drawn increasingly back, but reaching beyond rear tergal margin on segments 13–18 (Figure 9A–C).

**Figure 10.**
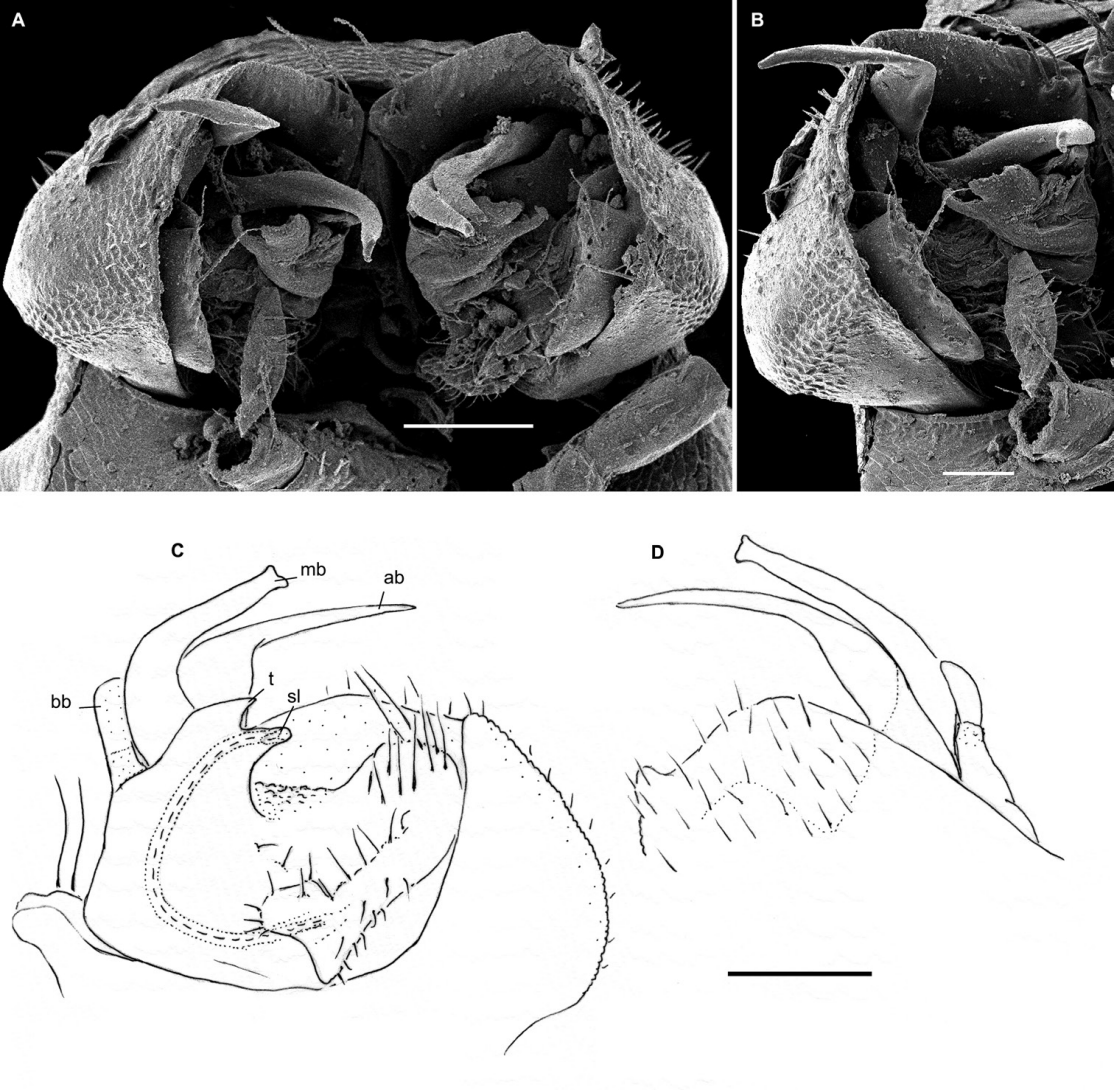
*Hemisphaeropariaongot* sp. n. **A** ♂ paratype, SEM micrographs of both gonopods in situ, caudoventral view **B** ♂ paratype, SEM micrographs of right gonopod, caudoventral view **C, D** left gonopod of holotype, mesal and lateral views, respectively. Scale bars: 0.1 mm (**A, C, D**), 0.05 mm (**B**). Abbreviations: **ab** apical branch of telopodite, **bb** basal branch of telopodite, **lo** lobe, **mb** medial branch of telopodite, **sl** solenomere, **t** tooth.

Gonopodal telopodite (Figure 10) deeply sunken inside a deep gonocoel, densely setose at base with three main branches (ab, mb, bb) mostly exposed: bb the shortest and rounded on top, mb slightly curved and subtruncate, and ab the longest and also slightly curved. Seminal groove relatively long, ending on a short solenomere (sl) supplied with an evident tooth (t) at base of sl and ab.

### Hemisphaeroparia digitifer sp. n.

Taxon classificationAnimaliaPolydesmidaTrichopolydesmidae

http://zoobank.org/321E3C2F-1DB7-4A04-9404-A6DF51B47817

Figs 11 , 12 , 28E

#### Type material.

Holotype ♂ (MRAC 22751), Cameroon, Littoral Region, Nkam Division, Koukoe, forest, 04°08’N, 010°10’E, 28.IX.2017, leg. A.R. Nzoko Fiemapong and J.A. Yetchom Fonjo.

Paratypes: 1 ♂ (SEM, MRAC 22752), 2 ♂♂ (with one gonopod retained in situ)(MRAC 22753), same locality, 28.IX.2017, leg. A.R. Nzoko Fiemapong and J.A. Yetchom Fonjo.

#### Diagnosis.

Differs from all other species of the genus by the presence of a boletiform epicranial tubercle (♂), coupled with the gonopodal telopodites that are deeply sunken inside a large gonocoel and show three main branches (ab, mb, bb), all exposed in their distal parts and followed by no lobe, but instead with a conspicuous, setose, fully concealed finger (d) basally in apical part; seminal groove relatively long and straight, ending subapically on ab without any trace of a solenomere (Figure 12).

#### Name.

To emphasize the presence of a conspicuous, setose, fully concealed finger (d) on the gonopodal telopodite; noun in apposition.

#### Description.

Length of holotype ca. 4 mm (♂), width of midbody pro- and metazonae 0.3 and 0.45 mm (♂), respectively. Length of paratypes ca. 5 mm, width of midbody pro- and metazonae 0.45–0.6. Coloration in alcohol light yellow (Figure 28E).

All other characters as in *H.zamakoe* sp. n., except as follows.

**Figure 11.**
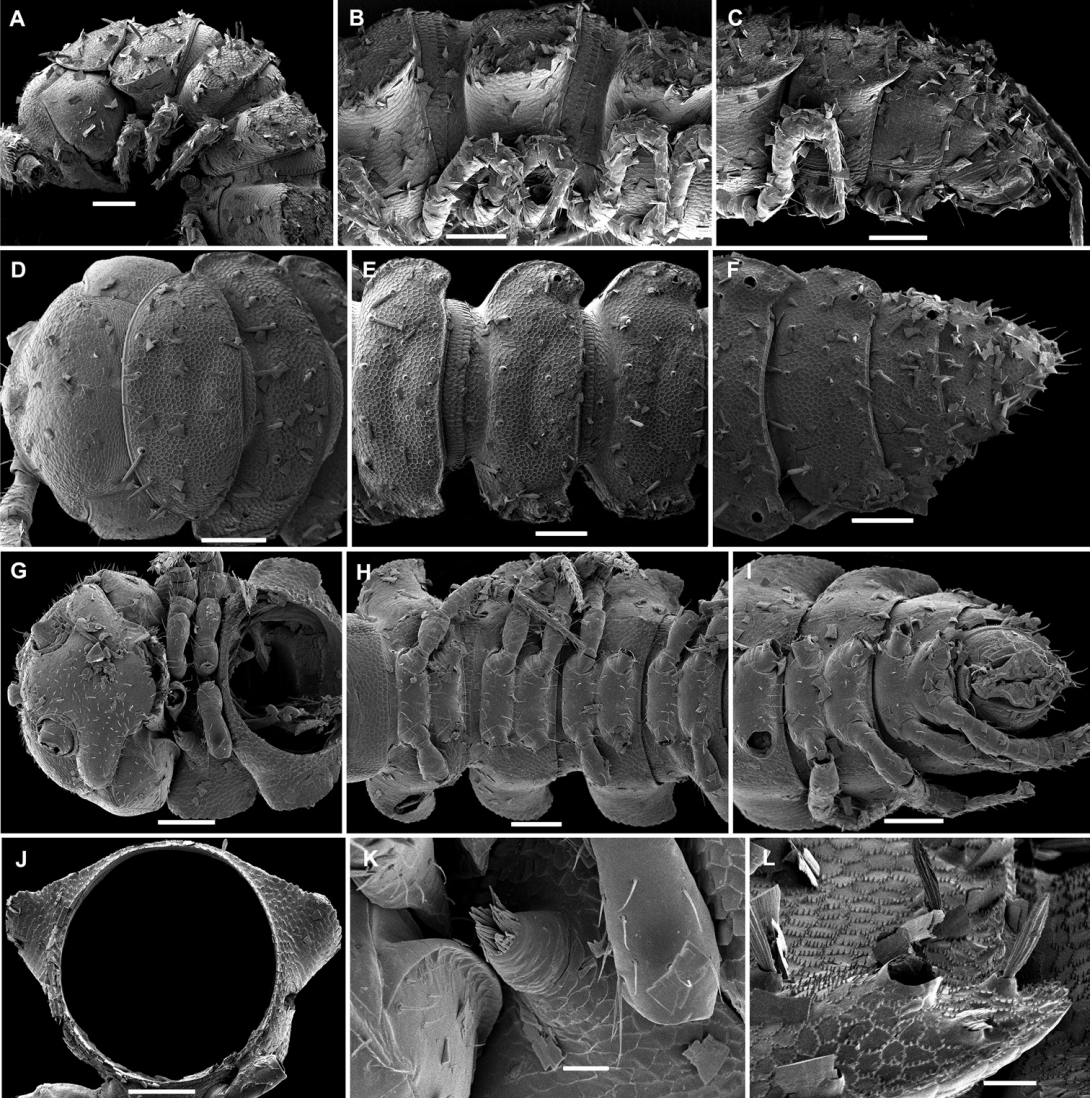
*Hemisphaeropariadigitifer* sp. n., SEM micrographs of ♂ paratype **A, D, G** anterior part of body, lateral, dorsal and ventral views, respectively **B, E, H** midbody segments, lateral, dorsal and ventral views, respectively **C, F, I** posterior part of body, lateral, dorsal and ventral views, respectively **J** midbody segment, caudal view **K** spiracle lateral to coxa 2 **L** midbody paratergum, lateral view. Scale bars: 0.1 mm (**A–J**), 0.02 mm (**K, L**).

Body with 20 segments. Antennae long and strongly clavate, reaching back to segment 3 when stretched dorsally (♂). Tergal setae mainly short, each often ca. 1/6–1/7 as long as metatergum, bacilliform, or subclavate (Figure 11A–F, L). A faint transverse sulcus often traceable between rows 1 and 2 of setae on some metaterga. Segment 2 with a prominent and apically complex spiracle on each side (Figure 11K).

Legs rather long and slender, ca. 1.2–1.3 times as long as midbody height (♂).

**Figure 12.**
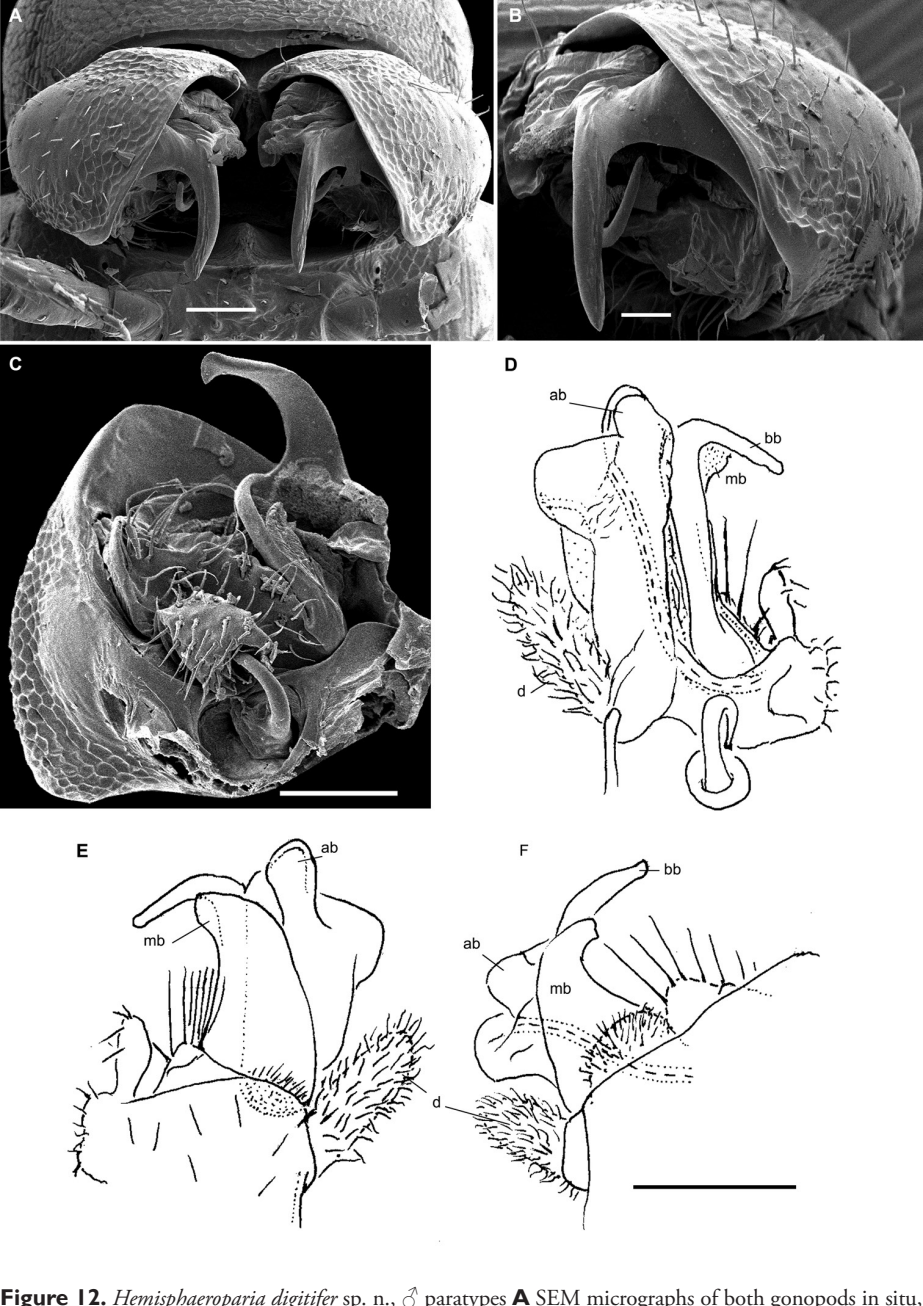
*Hemisphaeropariadigitifer* sp. n., ♂ paratypes **A**SEM micrographs of both gonopods in situ, ventral view **B, C**SEM micrographs of left gonopod, ventral view **D, E** right gonopod, mesal view **F** left gonopod, mesal and lateral views, respectively. Scale bars: 0.05 mm (**A–C**), 0.1 mm (**D–F**). Abbreviations: **ab** apical branch of telopodite, **bb** basal branch of telopodite, **d** setose finger, **mb** medial branch of telopodite.

Gonopods (Figure 12) with a deep gonocoel and complex, only little-exposed telopodites, the latter complex, showing subequally high ab and mb branches, the longest and most curved branch being bb. Apical part of telopodite with a conspicuous, long, and abundantly setose finger (d). Seminal groove long and straight, ending subapically on ab without any trace of a solenomere.

### Hemisphaeroparia parva sp. n.

Taxon classificationAnimaliaPolydesmidaTrichopolydesmidae

http://zoobank.org/9620E447-A2C0-4450-BD4C-9C3A869BBCFB

Figs 13 , 14 , 28F

#### Type material.

Holotype ♂ (MRAC 22754), Cameroon, Littoral Region, Sanaga Maritine Division, Mouanko, forest, 03°38’N, 009°46’E, leg. A.R. Nzoko Fiemapong and J.A. Yetchom Fonjo.

Paratypes: 4 ♂♂ (MRAC: 22755), 1 ♂ (SEM, MRAC 22756), 1 ♂ (ZMUM), 1 ♂ (UY1), same locality, together with holotype.

#### Diagnosis.

Differs from other species of the genus by having 19 body segments and by the absence of epicranial modifications in the ♂, coupled with the presence of only a single prominent branch (ab) which is exposed beyond the gonopodal coxa only distally; ab at the base with a large, lateral, finger-shaped process (lp); the seminal groove is rather long and moves onto a very short and retrose solenomere (sl) apically (Figure 14).

#### Name.

To emphasize the very small size; adjective in feminine gender.

#### Description.

Length of holotype ca. 3 mm (♂), width of midbody pro- and metazonae 0.25 and 0.3 mm, respectively. Length of paratypes 2.8–3.2 mm, width of midbody pro- and metazonae 0.2–025 and 0.3–0.4 mm, respectively (♂). Coloration in alcohol nearly pallid (Figure 28F), paratypes in places faintly pinkish.

All other characters as in *H.zamakoe* sp. n., except as follows.

**Figure 13.**
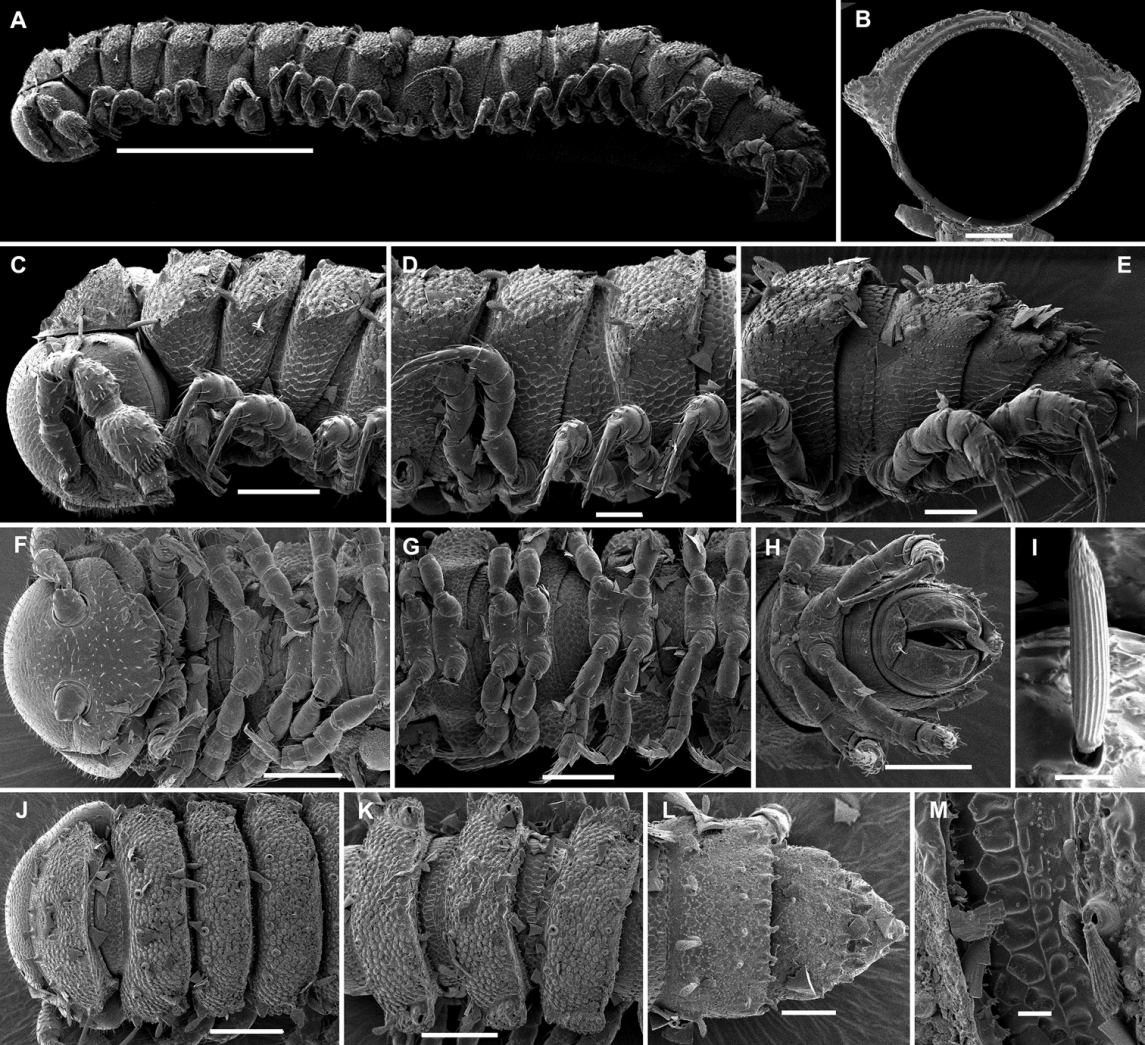
*Hemisphaeropariaparva* sp. n., SEM micrographs of ♂ paratype **A** habitus, lateral view **B** midbody segment, caudal view **C, F, J** anterior part of body, lateral, ventral and dorsal views, respectively **D, G, K** midbody segments, lateral, ventral and dorsal views, respectively **E, H, L** posterior part of body, lateral, ventral and dorsal views, respectively **I** tergal seta, lateral view **M** tergal fine structure. Scale bars: 0.5 mm (**A**), 0.1 mm (**C, F–H, J, K**), 0.05 mm (**B, D, E, L**), 0.01 mm (**I, M**).

Body with 19 (♂) segments. Head very densely micropilose, without epicranial modifications (♂)(Figure 13A, C, F, J). Interantennal isthmus ca. 1.5 times as large as diameter of antennal socket (Figure 13F). Antennae long and strongly clavate, reaching back to segment 3 when stretched dorsally (♂). In width, collum < segment 3 = 4 < 2 < head = 5–16; thereafter body gradually tapering towards telson. Collum and most of postcollum metaterga with three transverse regular rows of setae, but some metaterga in anterior body half without middle row (Figure 13C, D, J, K). Tergal setae short, bacilliform to slightly subclavate, longitudinally finely ribbed (Figure 13C–E, I-L). Paraterga always regularly declivous. Caudal corner of paraterga always rounded, never drawn back behind rear tergal margin (Figure 13C–E, J–L).

Legs rather long and slender, ca. 1.2–1.3 times as long as midbody height; in length, tarsus > femur > prefemur> coxa = postfemur = tibia (Figure 13D).

**Figure 14.**
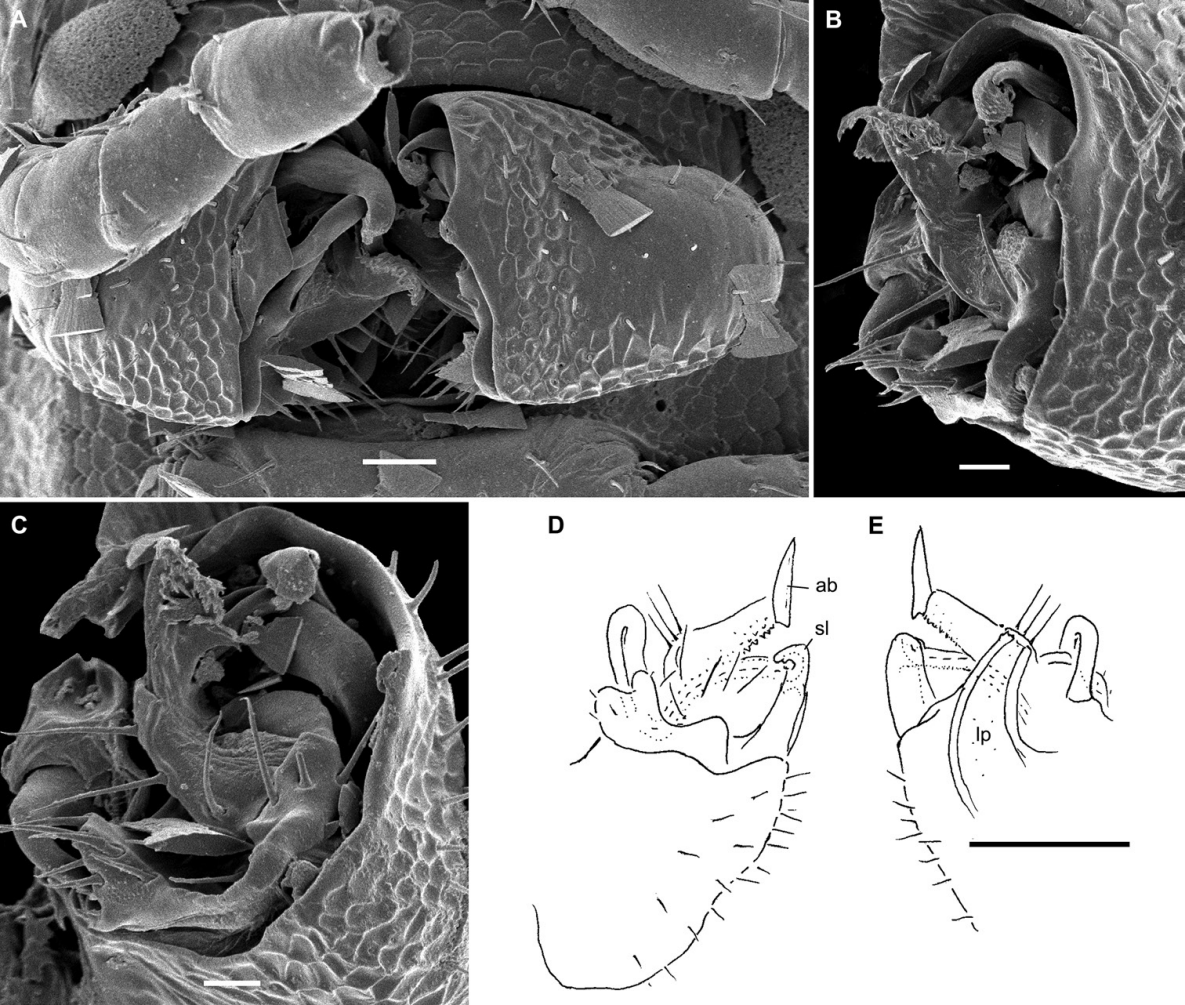
*Hemisphaeropariaparva* sp. n., ♂ paratypes **A**SEM micrographs of both gonopods in situ, ventral view **B, C**SEM micrographs of right gonopod, subcaudal and submesal views, respectively **D, E** right gonopod, mesal and lateral views, respectively. Scale bars: 0.1 mm (**D, E**), 0.02 mm (**A**), 0.01 mm (**B–C**). Abbreviations: **ab** apical branch of telopodite, **lp** lateral process, **sl** solenomere.

Gonopodal telopodite (Figure 14) almost fully concealed inside a deep gonocoel, with only a single prominent branch (ab), this being exposed beyond coxa only distally; ab at base with a large lateral process (lp). Seminal groove rather long, moving onto a very short and retrose solenomere (sl) apically.

### Hemisphaeroparia fusca sp. n.

Taxon classificationAnimaliaPolydesmidaTrichopolydesmidae

http://zoobank.org/430155E2-B5B3-4DF9-889A-589BABD13839

Figs 15 , 16 , 29A

#### Type material.

Holotype ♂ (MRAC 22757), Cameroon, Littoral Region, Nkam Division, Koukoe, forest, 04°08’N, 010°10’E, 28.IX.2017, leg. A.R. Nzoko Fiemapong and J.A.Yetchom Fonjo.

Paratypes: 1 ♂ (without gonopods)(MRAC 22758), 1 ♂ (SEM, MRAC 22759), same locality, 19.IV.2014; same locality, 21.III.2015; 1 adult ♀, 1 subadult ♀, 2 fragmented juveniles (MRAC 22760), same locality, together with holotype, all leg. A.R. Nzoko Fiemapong and J.A. Yetchom Fonjo.

#### Diagnosis.

Differs from all species of the genus by the absence of epicranial modifications (♂), coupled with the clearly more strongly developed and caudally acute paraterga compared to *H.zamakoe* sp. n. and most other congeners, as well as the presence of three prominent branches (ab, mb and bb) and a low lobe (lo) on the gonopodal telopodite and of a conspicuous foramen (fo) in the lateral wall to accommodate the end of the solenomere (sl) in a kind of pulvillus (Figure 16).

#### Name.

To emphasize the mostly dark coloration; adjective in feminine gender.

#### Description.

Length of holotype ca. 8.5 mm (♂), width of midbody pro- and metazonae 0.8 and 1 mm (♂), respectively. Length of paratypes (♀) 9.5 mm, width of midbody pro- and metazonae 0.9 and 1.2 respectively. Coloration in alcohol brown; head, antennae, gonopods, and venter light brown; legs light yellow-brown (Figure 29A). All type material badly overgrown with molds.

All other characters as in *H.zamakoe* sp. n., except as follows.

**Figure 15.**
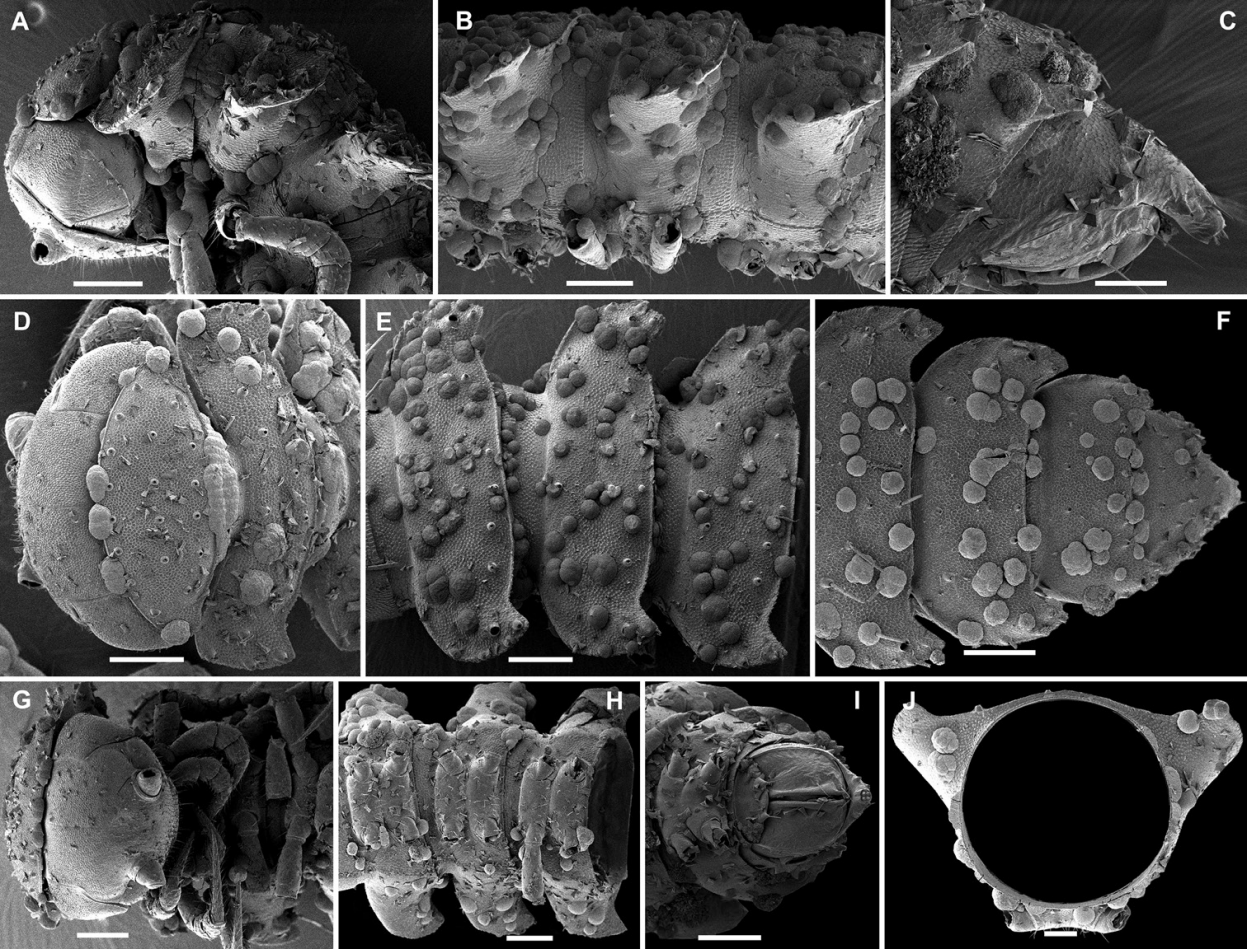
*Hemisphaeropariafusca* sp. n., SEM micrographs of ♂ paratype **A, D, G** anterior part of body, lateral, dorsal and ventral views, respectively **B, E, H** midbody segments, lateral, dorsal and ventral views, respectively **C, F, I** posterior part of body, lateral, dorsal and ventral views, respectively **J** midbody segment, caudal view. Scale bars: 0.2 mm (**A, B, D–I**), 0.1 mm (**C, J**).

Head without epicranial modifications (Figure 15A, D, G). Antennae long, slightly clavate, reaching back to segment 3 (♂) or 2 (♀) when stretched dorsally. In length, antennomere 3 > 2 = 5 = 6 > 4 > 7 > 1; antennomeres 5 and 6 the largest. In width, collum < head < segment 2 < 3 = 4 < 5–17. Collum ellipsoid, lateral angles acute and narrowly rounded. Tergal setae relatively long, bacilliform, mainly 1/3 as long as metatergum (Figure 29A). Dorsum nearly flat. Paraterga well-developed, set high, at around upper 1/4 of metazonae, mostly slightly upturned caudad (Figure 15A, F, J). Caudal corner of paraterga increasingly well drawn behind towards telson, narrowly rounded until around midbody segments, thereafter acute and drawn behind rear tergal margin, sharp and subspiniform (♂, ♀)(Figure 15A–F). Pleurosternal carinae traceable as a thin line on all segments.

Legs rather long and slender, ca. 1.3–1.4 (♂) or 1.1–1.2 (♀) times as long as midbody height; in length, tarsus > femur > prefemur> coxa = postfemur = tibia.

**Figure 16.**
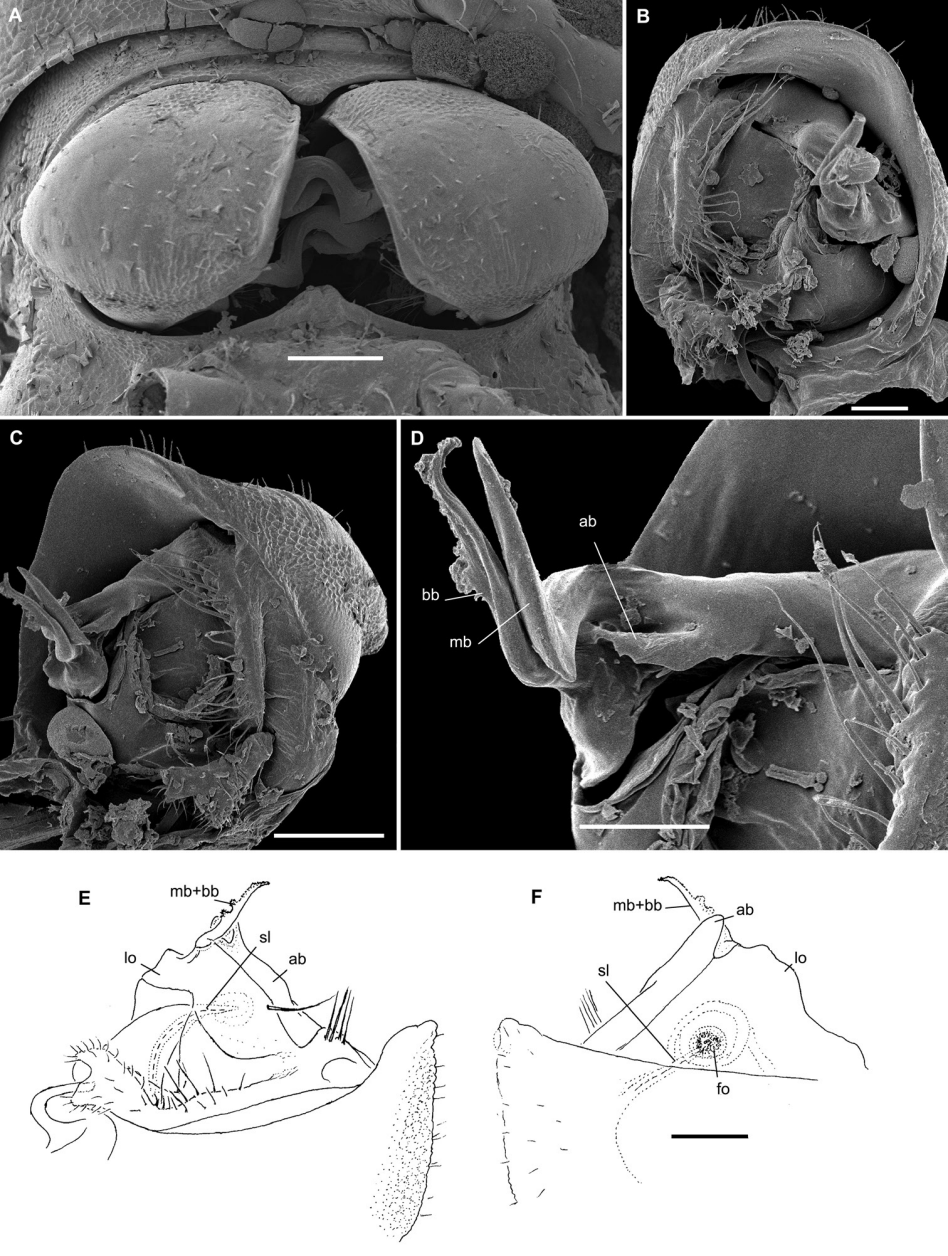
*Hemisphaeropariafusca* sp. n., ♂ paratype **A**SEM micrographs of both gonopods in situ, ventral view **B–D**SEM micrographs of left gonopod, submesal, ventromesal and enlarged ventromesal views, respectively **E, F** left gonopod of holotype (branch bb fully concealed), mesal and lateral views, respectively. Scale bars: 0.1 mm (**A, C, E, F**), 0.05 mm (**B, D**). Abbreviations: **ab** apical branch of telopodite, **fo** foramen, **lo** lobe, **mb+bb** medial and basal branches, **sl** solenomere.

Gonopodal telopodites (Figure 16) almost fully concealed inside a large gonocoel, with three branches (ab, mb, bb), all contiguous and moderately only exposed beyond coxa, followed by a small round lateral lobe (lo) more basally. Seminal groove short, moving onto a short subspiniform solenomere (sl), the latter subtransverse and directed laterad, and perforating the lateral wall to form a conspicuous foramen (fo) resembling a pulvillus because of numerous microscopic transparent filaments around.

### Hemisphaeroparia bonakanda sp. n.

Taxon classificationAnimaliaPolydesmidaTrichopolydesmidae

http://zoobank.org/1B374C62-B661-45D0-9007-DA2A8638BD71

Figs 17 , 18 , 29B

#### Type material.

Holotype ♂ (MRAC 22761) Cameroon, South West Region, Bonakanda, VHF trade, Mt Cameroon National Park, savannah, near edge of mountain forest, 04°13’53’’N, 009°15’19’’E, by hand, 19.X.2014, leg. K. Maes.

1 ♂ (lost), same locality, together with holotype.

#### Diagnosis.

Differs from other species of the genus by the presence of a boletiform epicranial tubercle (♂), coupled with relatively long tergal setae, line-shaped and microgranulate pleurosternal carinae, as well as deeply sunken gonopodal telopodites, each of which shows a single, moderately exposed, main branch (ab) and a rather short, subtransverse, laterally directed solenomere (sl), with a sharp tooth at its base (Figure 18).

#### Name.

To emphasize the type locality; noun in apposition.

#### Description.

Length of holotype ca. 7 mm (♂), width of midbody pro- and metazonae 0.55 and 0.8 mm (♂), respectively. Coloration light marbled grey-brown; head, antennae, legs, and venter light yellow-brown (Figure 29B).

All other characters as in *H.zamakoe* sp. n., except as follows.

**Figure 17.**
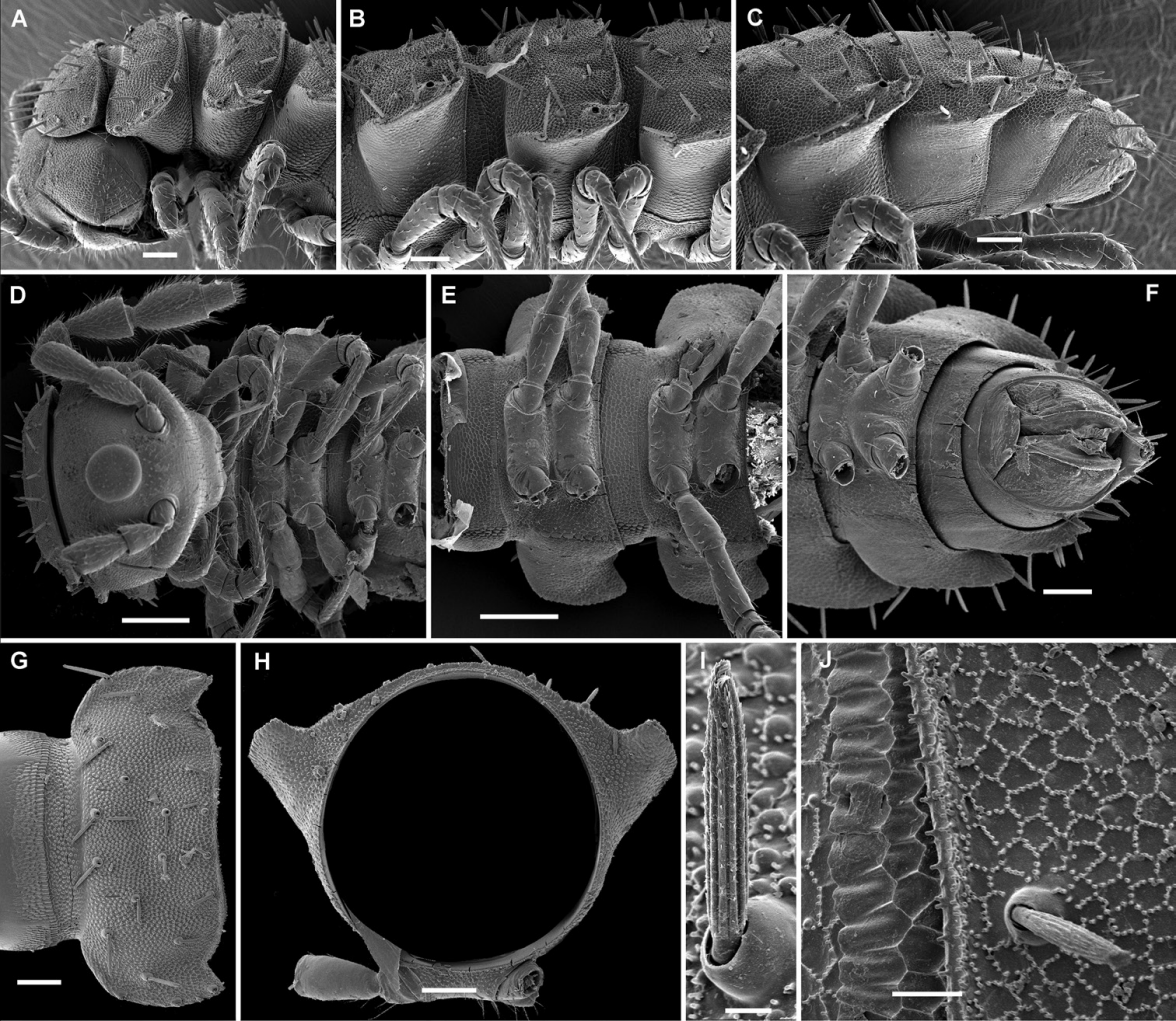
*Hemisphaeropariabonakanda* sp. n., SEM micrographs of ♂ paratype **A, D** anterior part of body, lateral and dorsal views, respectively **B, E** midbody segments, lateral and dorsal views, respectively **C, F** posterior part of body, lateral and dorsal views, respectively **G, H** midbody segment, dorsal and caudal view, respectively **I** tergal seta, lateral view **J** tergal fine structure, dorsal view. Scale bars: 0.2 mm (**D, E**), 0.1 mm (**A–C, F–H**), 0.02 mm (**J**), 0.01 mm (**I**).

Antennae long and strongly clavate, reaching behind to segment 3 when stretched dorsally (♂). In width, collum < 3 < 2 = 4 < 5–17; thereafter body gradually tapering towards telson. Tergal setae longer, each mostly ca. 1/4 as long as metatergum, bacilliform and ribbed (Figure 17). Caudal corner of paraterga always rounded, drawn increasingly back, but reaching beyond rear tergal margin only on segments 16–18 (Figs 17A–C, G). Limbus much more sparsely microspiculate (Figure 17J). Pleurosternal carinae visible on all segments as a thin granulated line (Figs 17B, C).

Legs rather long and slender, ca. 1.3–1.4 times as long as midbody height; in length, tarsus > femur > prefemur> coxa = postfemur = tibia.

**Figure 18.**
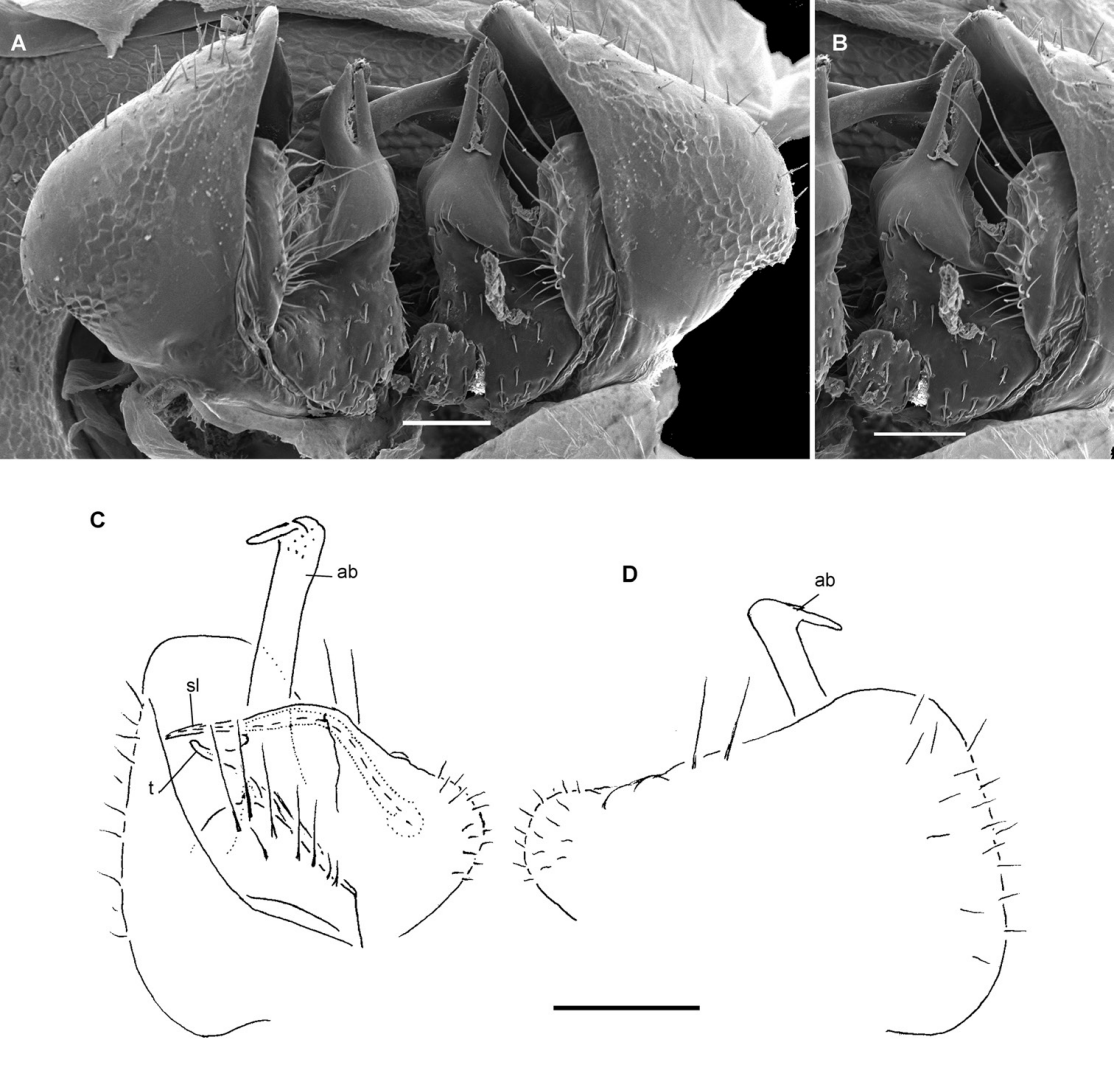
*Hemisphaeropariabonakanda* sp. n., ♂ paratype **A**SEM micrographs of both gonopods in situ, ventral view **B**SEM micrographs of right gonopod, ventral view **C, D** left gonopod of holotype, mesal and lateral views, respectively. Scale bars: 0.1 mm (**C, D**), 0.05 mm (**A, B**). Abbreviations: **ab** apical branch of telopodite, **sl** solenomere, **t** tooth.

Gonopodal telopodites (Figure 18) almost fully concealed inside a large gonocoel, with only one unequally bifid branch (ab) moderately strongly exposed beyond coxa. Seminal groove short, moving onto a short, subspiniform solenomere (sl), the latter subtransverse and directed laterally, with a short tooth (t) at base.

### Hemisphaeroparia bamboutos sp. n.

Taxon classificationAnimaliaPolydesmidaTrichopolydesmidae

http://zoobank.org/23364EA5-AE07-4F2A-B54B-6630EAA81D6C

Figs 19 , 20 , 29C

#### Type material.

Holotype ♂ (MRAC 22762) , Cameroon, West Region, Bamboutos Division, Babajou District, Mt Bamboutos, 5°41’5”N, 10°06’23”E, 2600 m a.s.l., forest, 03.III.2017, leg. A.R. Nzoko Fiemapong.

Paratypes: 1 ♂ (SEM, MRAC 22763), same locality, together with holotype; 4 ♀♀ (MRAC 22764), same locality, 17.X.2017, all leg. A.R. Nzoko Fiemapong.

#### Diagnosis.

Differs from other species of the genus by the presence of a large, round, epicranial bulge (♂), coupled with the gonopodal telopodites being only moderately exposed above a deep gonocoel, each telopodite with only two branches (ab, bb), contiguous and followed by a small rounded lobe (lo) more basally. Branch bb shorter, with a conspicuous distomesal tooth (t). Seminal groove short, ending on a short retrorse solenomere (sl) nearly level with bb tip.

#### Name.

To emphasize the type locality; noun in apposition.

#### Description.

Length of holotype ca. 4.5 mm (♂), width of midbody pro- and metazonae 0.5 and 0.7 mm (♂), respectively. Length of ♀ paratypes 4–5 mm, width of midbody pro- and metazonae 0.4 –0.65 and 0.6–0.8 mm, respectively. Coloration in alcohol nearly pallid (♂, ♀)(Figure 29C).

All other characters as in *H.zamakoe* sp. n., except as follows.

**Figure 19.**
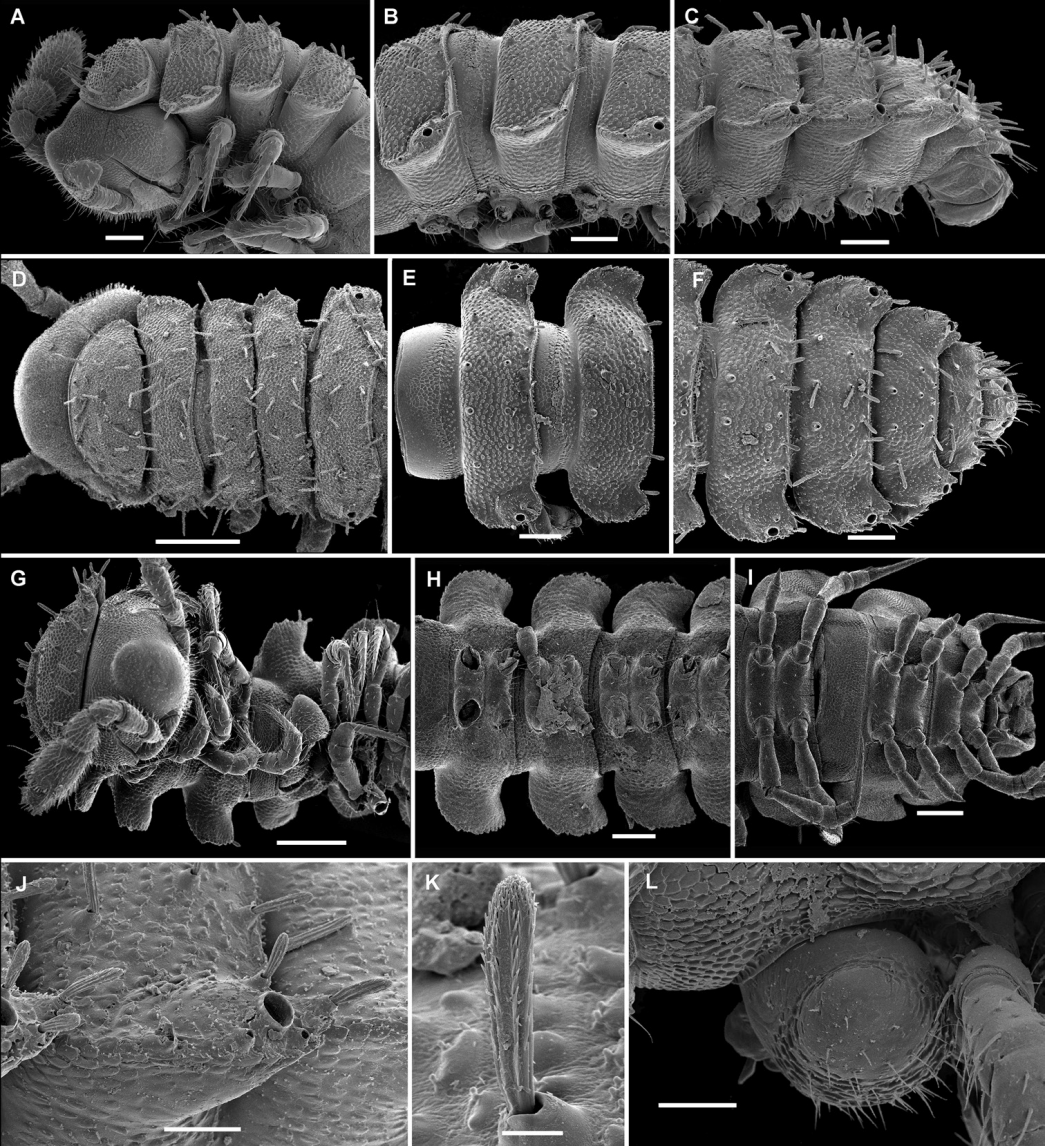
*Hemisphaeropariabamboutos* sp. n., SEM micrographs of ♂ paratype **A, D, G** anterior part of body, lateral, dorsal and ventral views, respectively **B, E, H** midbody segments, lateral, dorsal and ventral views, respectively **C, F, I** posterior part of body, lateral, dorsal and ventral views, respectively **J** midbody paratergum, lateral view **K** tergal seta, lateral view **J** midbody segment, caudal view **L** gonopods in situ, lateral view. Scale bars: 0.2 mm (**D, G, I**), 0.1 mm (**A–C, E, F, H**), 0.05 mm (**J, L**), 0.01 mm (**K**).

Body with 19 segments (♂, ♀). Head with a large, round, densely micropilose, epicranial bulge (♂)(Figs 19A, D, G). Antennae long and clavate, reaching behind to segment 3 when stretched dorsally (♂, ♀). In length, antennomere 3 = 6 < 2 = 5 < 4 = 7 < 1 (♂, ♀). In width, collum < 3 < 2 = 4 < head < 5–15; thereafter body gradually tapering towards telson. Tergal setae slightly longer, each ca. 1/3–1/4 as long as metatergum, slightly clavate and bacilliform, ribbed and extremely delicately serrate (Figure 19K), setation pattern rather irregular per row: 2+2–4+4, 2+2–3+3 and 3+3–4+4 in rows 1–3, respectively. Caudal corner of paraterga always rounded, drawn increasingly back, but reaching beyond rear tergal margin only on segments 14–17 (Figs 19A–F). Pleurosternal carinae a very delicately granulate line visible on all segments (Figure 19A–C).

Legs rather long and slender, ca. 1.3–1.4 (♂) or 1.1–1.2 (♀) times as long as midbody height.

**Figure 20.**
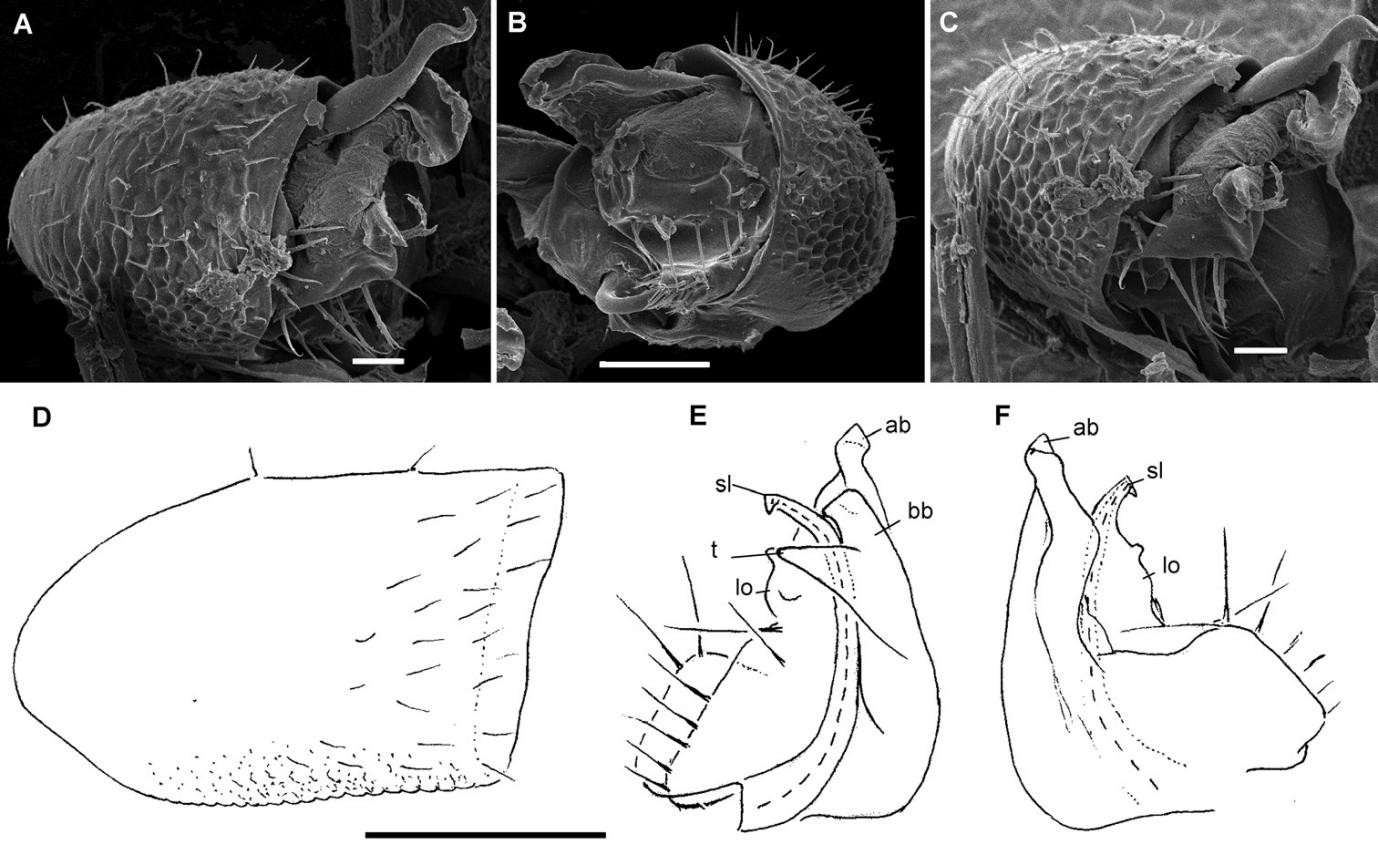
*Hemisphaeropariabamboutos* sp. n., ♂ paratype **A, C**SEM micrographs of right gonopod, lateral and ventrolateral views, respectively B left gonopod, ventromesal view **D–F** right gonopod of holotype, coxa (**D**) and telopodite (**E, F**), ventral, mesal and lateral views, respectively. Scale bars: 0.1 mm (**D–F**), 0.05 mm (**B**), 0.02 mm (**A, C**). Abbreviations: **ab** apical branch of telopodite, **bb** basal branch of telopodite **lo** lobe, **sl** solenomere, **t** tooth.

Gonopodal telopodites (Figure 20) only moderately exposed above a deep gonocoel, each with only two branches (ab, bb), both being contiguous and followed by a small rounded lobe (lo) more basally. Branch bb shorter, with a conspicuous distomesal tooth (t). Seminal groove short, ending on a short retrorse solenomere (sl) nearly level with bb tip.

### Hemisphaeroparia subfalcata sp. n.

Taxon classificationAnimaliaPolydesmidaTrichopolydesmidae

http://zoobank.org/D0F4DA21-564C-485D-9D90-756DA0A667ED

Figs 21 , 22 , 29D

#### Type material.

Holotype ♂ (MRAC 22765), Cameroon, Center Region, Yaounde I University campus, palm plantation, 03°53’N, 011°30’E, 860 m a.s.l., 20.III.2018, leg. A.R. Nzoko Fiemapong.

Paratypes: 1 ♂ (SEM, MRAC 22766), 1 ♂ (without gonopods)(MRAC 22767), same locality, together with holotype; 4 ♂♂, 1 ♀ (MRAC 22768), 1 ♂ (ZMUM), 1 ♂ (UY1), same locality, 7.IV.2014, all leg. A.R. Nzoko Fiemapong.

#### Diagnosis.

Differs from other species of the genus by the presence of a boletiform epicranial tubercle (♂) and 19 segments in both sexes, coupled with the gonopodal telopodites (Figure 22) showing only one, but especially prominent and subfalcate main branch (ab), this being very strongly exposed beyond coxa; a long spiniform solenomere (sl) is subtransverse, directed forward and shows a short truncated tooth (t) at its base.

#### Name.

To emphasize the subfalcate gonopodal branch ab; adjective in feminine gender.

#### Description.

Length of holotype ca. 3.8 mm (♂), width of midbody pro- and metazonae 0.35 and 0.5 mm (♂), respectively. Length of ♂ paratypes 3–3.4 mm, width of midbody pro- and metazonae 0.25–0.35 and 0.5–0.7 mm, respectively; ♀ paratype 3.5 mm long, 0.25 and 0.4 mm wide on midbody pro- and metazonae, respectively. Coloration of holotype in alcohol light marbled brown; legs, head, and venter light brown-yellow. All paratypes lighter, light grey-brown to nearly pallid (Figure 29D).

All other characters as in *H.zamakoe* sp. n., except as follows.

**Figure 21.**
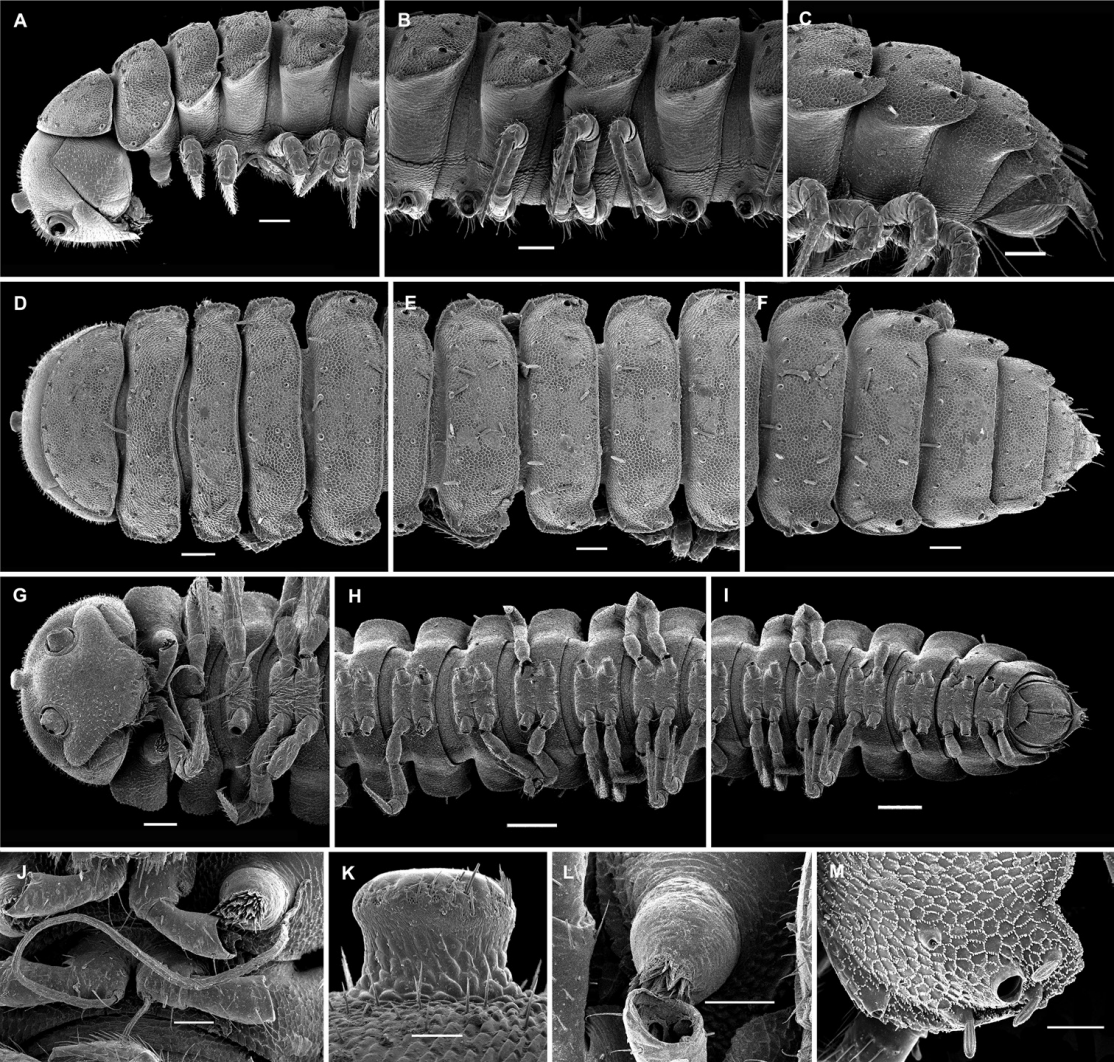
*Hemisphaeropariasubfalcata* sp. n., SEM micrographs of ♂ paratype **A, D, G** anterior part of body, lateral, dorsal and ventral views, respectively **B, E, H** midbody segments, lateral, dorsal and ventral views, respectively **C, F, I** posterior part of body, lateral, dorsal and ventral views, respectively **J, L** spiracle lateral to coxa 2, lateral and sublateral views, respectively **K** epicranial tubercle, dorsal view **M** midbody paratergum, lateral view. Scale bars: 0.2 mm (**H, I**), 0.1 mm (**A–G**), 0.05 mm (**J, M**), 0.02 mm (**K, L**).

Body with 19 segments (♂, ♀). In width, collum < 3 = 4 < 2 < head < segments 5–17; thereafter body gradually tapering towards telson. Caudolateral corner of collum acute and very norrowly rounded. Tergal setae medium-sized, bacilliform, usually a little longer, each 1/4–1/2 times as long as metatergum (Figs 21D–F), always 3+3 in each row on postcollum metaterga. Paraterga medium-sized, set at ca. 1/3 of upper 1/3 of metazonae (Figs 21A–C), visible starting with collum, mostly slightly declivous to subhorizontal often slightly upturned caudally, faintly, but regularly rounded and bordered, lateral incisions absent. Caudal corner of paraterga always rounded, drawn increasingly back, but reaching beyond rear tergal margin only on segments 16 and 17 (Figs 21C, F). Segment 2 with a very prominent, boletiform and apically complex spiracle on each side (Figs 21A, J, L).

**Figure 22.**
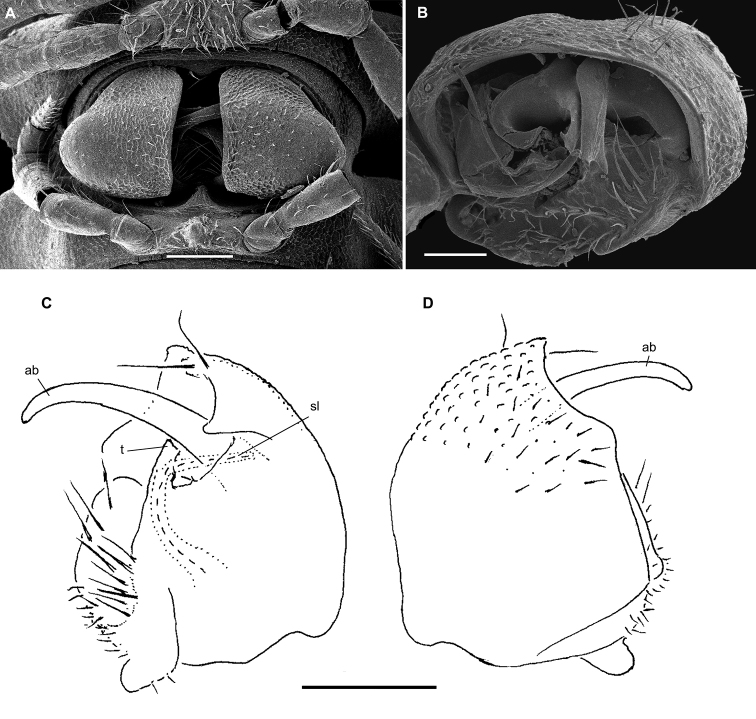
*Hemisphaeropariasubfalcata* sp. n., ♂ paratypes **A**SEM micrographs of both gonopods in situ, ventral view **B**SEM micrographs of left gonopod, submesal view **C, D** right gonopod, mesal and lateral views, respectively. Scale bars: 0.1 mm (**A, C, D**), 0.05 mm (**B)**. Abbreviations: **ab** apical branch of telopodite, **sl** solenomere, **t** tooth.

Legs rather long and slender, ca. 1.3–1.4 (♂) or 1.1–1.2 (♀) times as long as midbody height. Sternum behind gonopods with a small central tubercle (Figure 22A).

Gonopodal telopodites (Figure 22) almost fully concealed inside a large gonocoel, each with only one main branch (ab), this being long, subfalcate, and very strongly exposed beyond coxa. Seminal groove short, at around midlength moving onto a long, spiniform solenomere (sl), the latter subtransverse, directed forward and showing a short truncated tooth (t) at base.

### Hemisphaeroparia falcata sp. n.

Taxon classificationAnimaliaPolydesmidaTrichopolydesmidae

http://zoobank.org/0D4301A6-F7DC-4D08-BBE6-D12541E96EF0

Figs 23 , 24 , 29E

#### Type material.

Holotype ♂ (MRAC 22769), Cameroon, Center Region, Awae, secondary forest, 03°06’N, 10°29’E, 27.III.2018, leg. A.R. Nzoko Fiemapong.

Paratypes: 1 ♂ (SEM, MRAC: 22770), 1 ♂ (incomplete, badly fragmented and without gonopods)(MRAC 22771), same locality, together with holotype.

#### Diagnosis.

**Figure 23.**
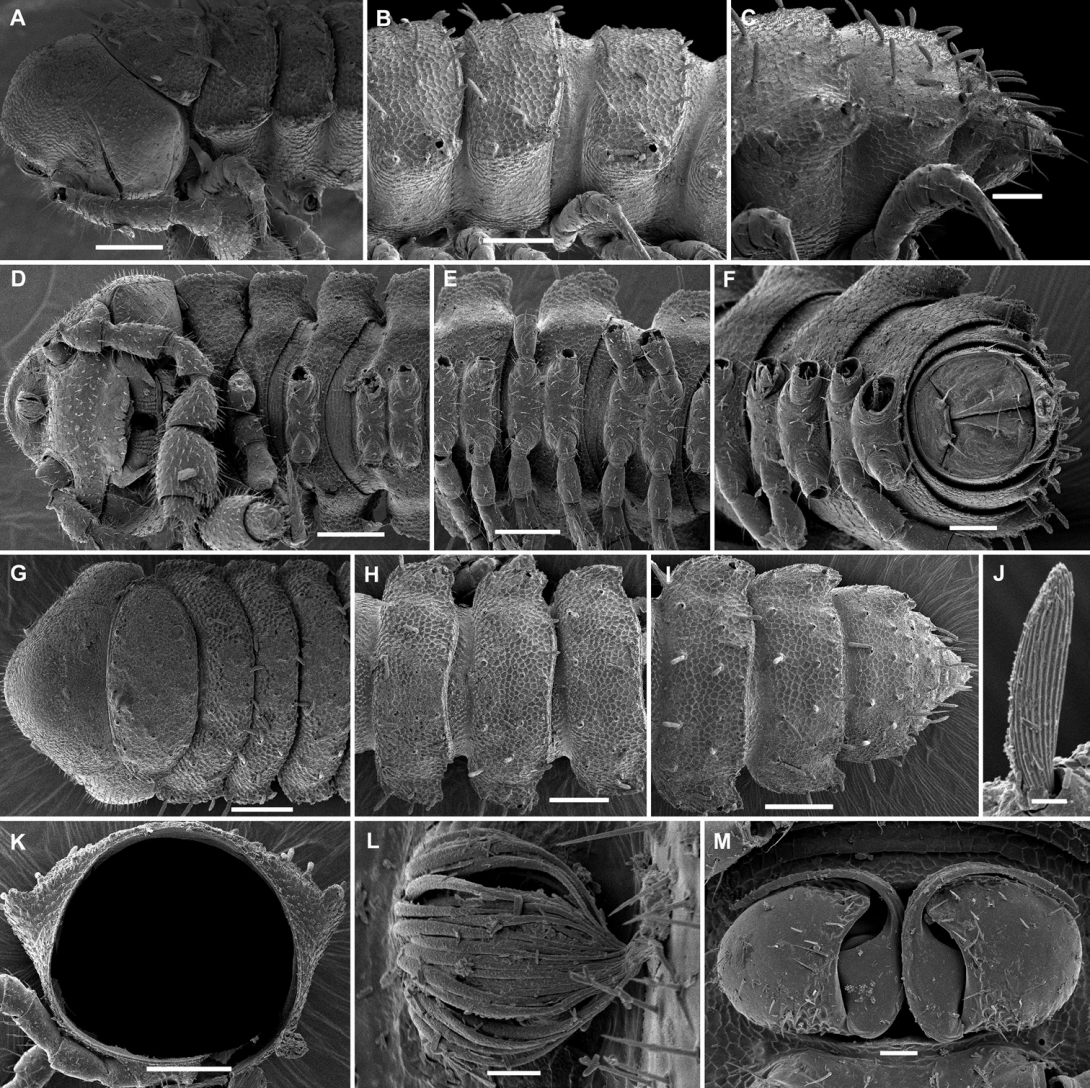
*Hemisphaeropariafalcata* sp. n., SEM micrographs of ♂ paratype **A, D, G** anterior part of body, lateral, ventral and dorsal views, respectively **B, E, H** midbody segments, lateral, ventral and dorsal views, respectively **C, F, I** posterior part of body, lateral, ventral and dorsal views, respectively **J** tergal seta, lateral view **K** midbody segment, caudal view **L** epicranial tubercle with filaments **M** both gonopods in situ, ventral view. Scale bars: 0.1 mm (**A, B, D, E, G–I, K**), 0.05 mm (**C, F**), 0.02 mm (**M**), 0.01 mm (**L**), 0.005 mm (**J**).

**Figure 24.**
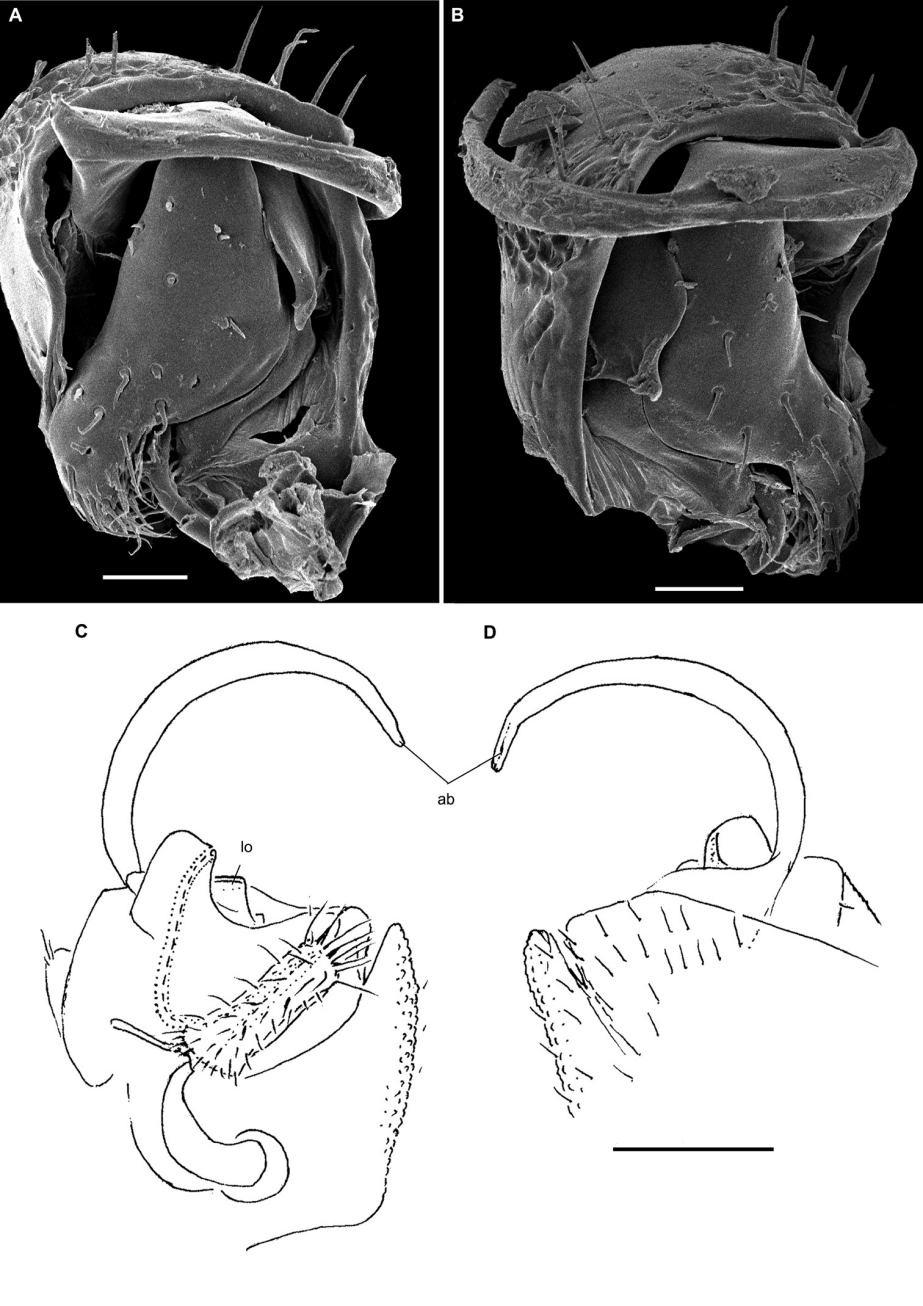
*Hemisphaeropariafalcata* sp. n., ♂ paratypes **A, B**SEM micrographs of left gonopod, ventromesal and submesal views, respectively **C, D** left gonopod, mesal and lateral views, respectively. Scale bars: 0.1 mm (**C, D**), 0.02 mm (**A, B**). Abbreviations: **ab** apical branch of telopodite, **lo** lobe.

Differs from other species of the genus by the presence of a particular epicranial tubercle with fine filaments on top (♂), of 19 body segments (♂), coupled with only one main branch (ab) on the gonopodal telopodite, this branch being very strongly exposed, very long, falcate and directed laterally, followed by a very small lobe (lo) more basally (Figs 23M, 24). No solenomere.

#### Name.

To emphasize the strongly falcate gonopodal branch ap; adjective in feminine gender.

#### Description.

Length of holotype ca. 3.5 mm (♂), width of midbody pro- and metazonae 0.25 and 0.4 mm (♂), respectively. Width of midbody pro- and metazonae of paratype 0.5 amd 0.7 mm, respectively. Coloration in alcohol light marbled red-brown, prozonae, le.g., and venter light grey-yellow (♂)(Figure 29E).

All other characters as in *H.zamakoe* sp. n., except as follows.

Body with 19 segments (♂). Epicranial region concave anteriorly in front of a clear swelling and bearing at bottom a conspicuous round tubercle supporting a tight group of numerous long filaments, these directed anteriorly (Figs 23D, L). Antennae relatively short and clavate, reaching behind to almost segment 3 when stretched dorsally (♂). In length, antennomere 3 = 6 > 5 > 2 = 4 = 7 > 1. In width, collum < segment 3=4 < 2 < head = 5–16; thereafter body gradually tapering towards telson. Tergal setae medium-sized to short, each ca. 1/4–1/3 as long as metatergum, bacilliform or subclavate, ribbed all along (Figs 23A–C, G–K). Paraterga medium-sized, set at around upper 1/3 of metazonae (Figs 23A–C, K), mostly declivous to subhorizontal, often slightly upturned caudally, faintly, but regularly rounded and bordered, lateral incisions absent. Caudal corner of paraterga always rounded, drawn increasingly back, but slightly reaching behind rear tergal margin only on segment 17 (Figs 23C, I).

Legs a little shorter (♂), slender, ca. 1.1–1.2 times as long as midbody height; in length, tarsus > femur > prefemur > coxa = postfemur = tibia.

Gonopodal telopodites (Figs 23M, 24) almost fully concealed inside a very large gonocoel, each with only one main branch (ab) very strongly exposed beyond coxa, being also unusually long, falcate and directed laterally, followed by a very small round lobe (lo) more basally. Seminal groove short, ending on a small squarish lobe without a solenomere.

### Hemisphaeroparia mouanko sp. n.

Taxon classificationAnimaliaPolydesmidaTrichopolydesmidae

http://zoobank.org/4C367275-8A1B-4AAC-95E9-1A503F1F279E

Figs 25 , 26 , 29F

#### Type material.

Holotype ♂ (MRAC 22772), Cameroon, Littoral Region, Sanaga Maritine Division, Mouanko, forest, 03°38’N, 009°46’E, 16.VIII.2017, leg. A.R. Nzoko Fiemapong and J.A. Yetchom Fonjo.

Paratypes: 2 ♂♂ (without gonopods)(MRAC 22773), 1 ♂ (SEM, MRAC 22774), same locality, together with holotype.

#### Diagnosis.

Differs from other species of the genus by 19 body segments (♂), the presence of a boletiform epicranial tubercle inside a depression (♂), coupled with each gonopodal coxa supplied with two unusually strong basal setae and the telopodites which are deeply sunken inside a large gonocoel and show only two, contiguous, little-exposed branches (ab, bb). The solenomere (sl) is long and finger-shaped (Figure 26).

#### Name.

To emphasize the type locality; noun in apposition.

#### Description.

Length of holotype and paratype ca. 3.5 mm (♂), width of midbody pro- and metazonae 0.2 and 0.35 mm (♂), respectively. Colouration in alcohol almost uniformly very light yellow brownish (Figure 29F).

All other characters as in *H.zamakoe* sp. n., except as follows.

**Figure 25.**
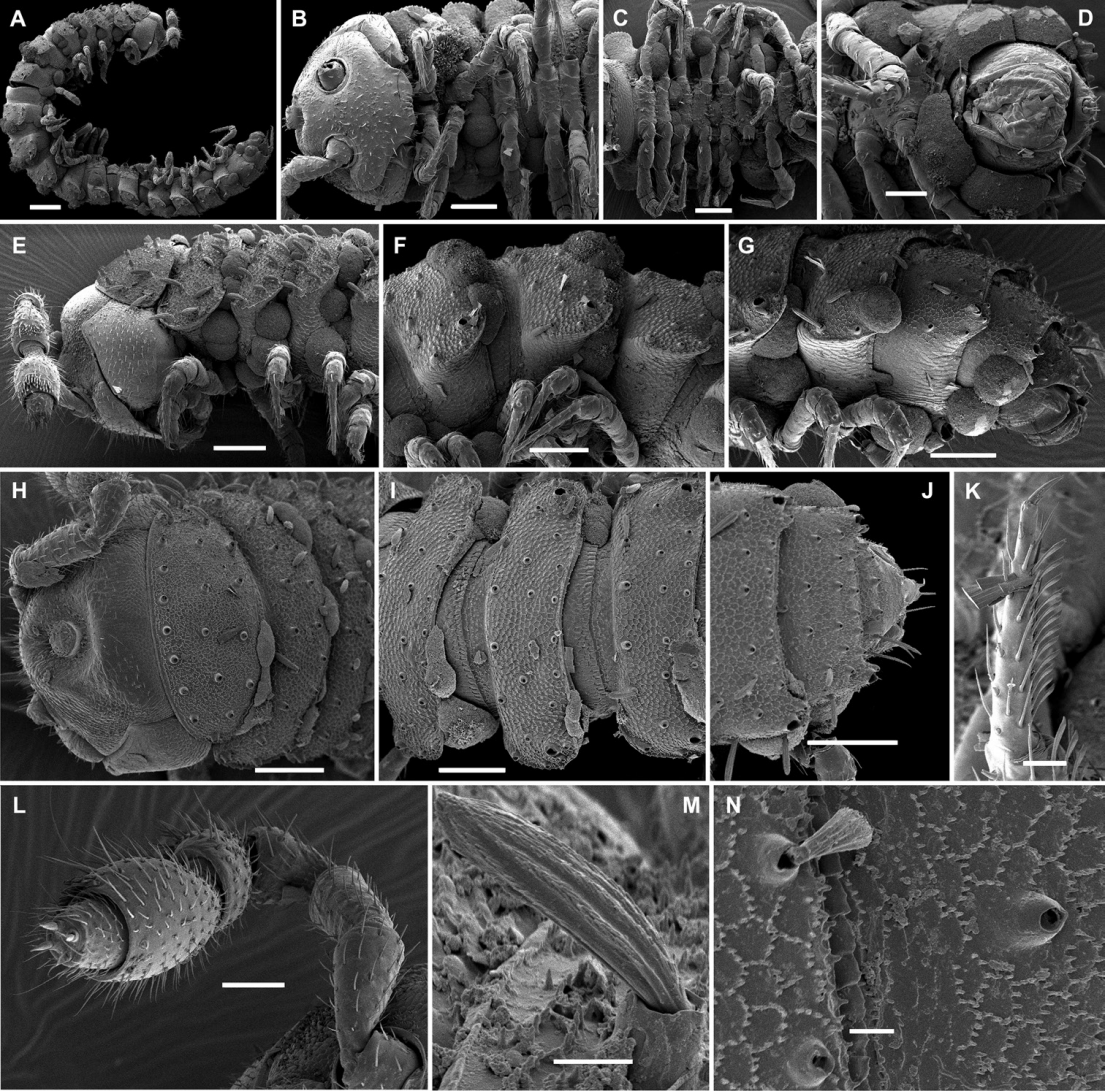
*Hemisphaeropariamouanko* sp. n., SEM micrographs of ♂ paratype **A** habitus, lateral view **B, E, H** anterior part of body, ventral, lateral and dorsal views, respectively **C, F, I** midbody segments, ventral, lateral and dorsal views, respectively **D, G, J** posterior part of body, ventral, lateral and dorsal views, respectively **K** tarsus from anterior part of body, lateral view **L** antenna, sublateral view **M** bacilliform tergal seta, lateral view **N** tergal fine structure with a subclavate seta, dorsal view. Scale bars: 0.2 mm (**A**), 0.1 mm (**B, C, E–J**), 0.05 mm (**D, L**), 0.02 mm (**K**), 0.01 mm (**M, N**).

Body with 19 segments (♂). Epicranial region with a boletiform tubercle at bottom of an excavation in front of an evident swelling (Figs 25B, H). Interantennal isthmus ca. 1.5 times as large as diameter of antennal socket. Antennae long and strongly clavate, reaching behind to segment 3 when stretched dorsally (♂). In length, antennomere 3 = 6 > 5 > 1 = 2 = 4 = 7. In width, collum < 2–4 < head = 5–15; thereafter body gradually tapering towards telson. Tergal setae medium-sized to short, each ca. 1/5–1/3 as long as metatergum, mostly bacilliform, more rarely subclavate, all ribbed (Figs 25M, N). Paraterga medium-sized, set at ca. upper 1/3 of metazonae), mostly regularly declivous (Figs 25A, E–G, I, J). Caudal corner of paraterga always rounded, drawn increasingly back, but reaching beyond rear tergal margin only on segments 16 and 17 (Figs 25J, M).

Legs rather long and slender, ca. 1.3–1.4 times as long as midbody height (♂); tarsi in anterior body half with ventral brushes (Figure 25K).

**Figure 26.**
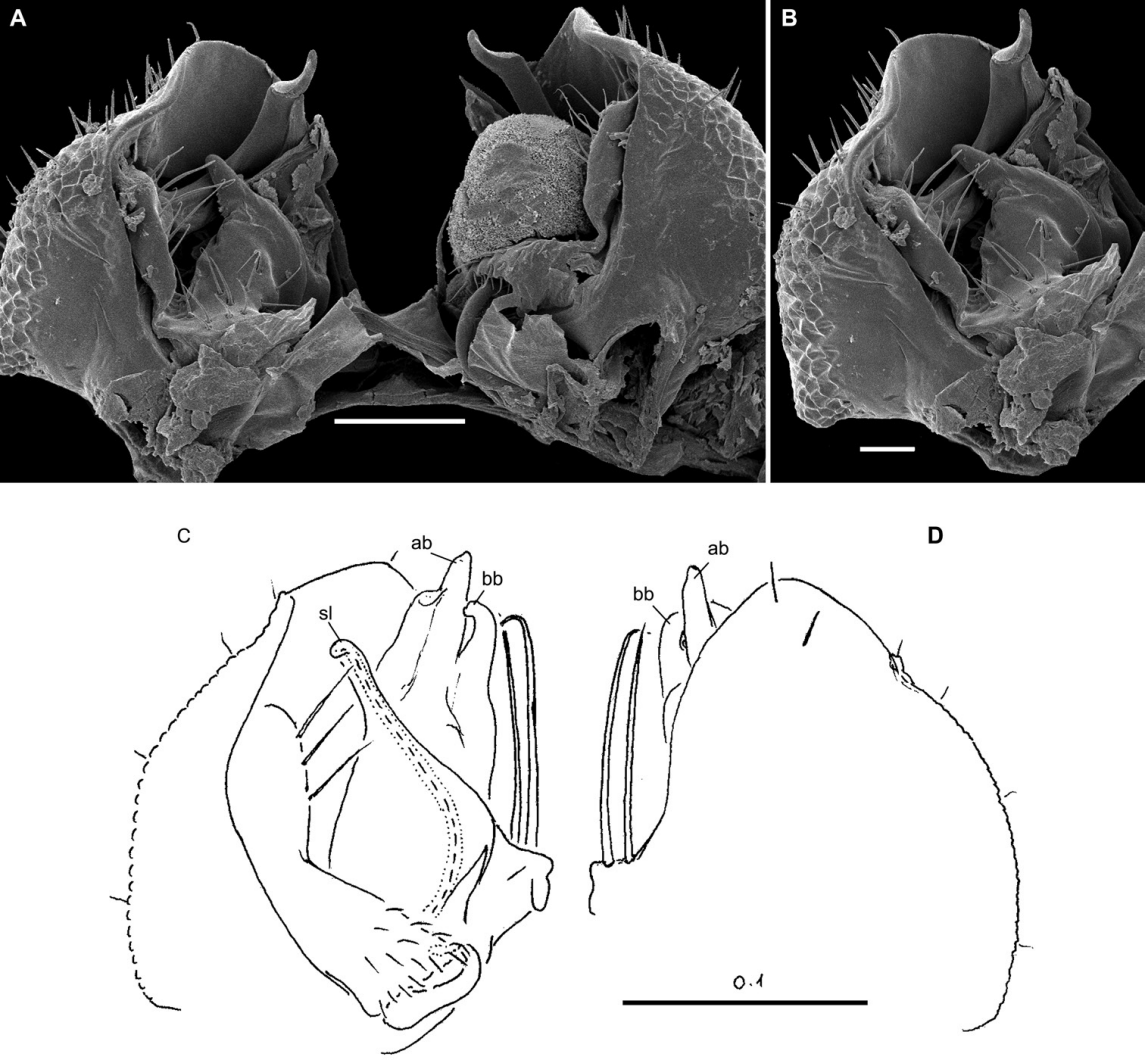
*Hemisphaeropariamouanko* sp. n., ♂ paratypes **A**SEM micrographs of both gonopods in situ, ventrocaudal view **B**SEM micrographs of right gonopod, ventrocaudal view **C, D** left gonopod, mesal and lateral views, respectively. Scale bars: 0.1 mm (**C, D**), 0.05 mm (**A**), 0.02 mm (**B**). Abbreviations: **ab** apical branch of telopodite, **bb** basal branch of telopodite, **sl** solenomere.

Gonopodal coxa with two unusually strong setae at base, at fusion site of both coxae (Figs 26C, D). Telopodite (Figure 26) almost fully concealed inside a very large gonocoel, each with only two branches (ab, bb), both contiguous and only slightly exposed beyond coxa, followed by no lobe more basally. Branch ab a little longer and faintly subdivided into two, branch bb shorter and slightly curved at tip. Seminal groove short, moving onto a long finger-shaped solenomere (sl).

### Hemisphaeroparia integrata

Taxon classificationAnimaliaPolydesmidaTrichopolydesmidae

(Porat, 1894) comb. n.

Figure 27

Polydesmus integratus Porat, 1894: 30 (original description).

#### Type material.

Lectotype ♂ (Naturhistoriska riksmuseet, Stockholm), Cameroon, leg. Y. Sjöstedt.

Paralectotypes: 2 fragmented ♀♀ (Naturhistoriska riksmuseet, Stockholm), together with lectotype.

One of us (JPM) revised the types and made the present lectotype designation, the latter to ensure that the species is based on male material.

#### Diagnosis.

**Figure 27.**
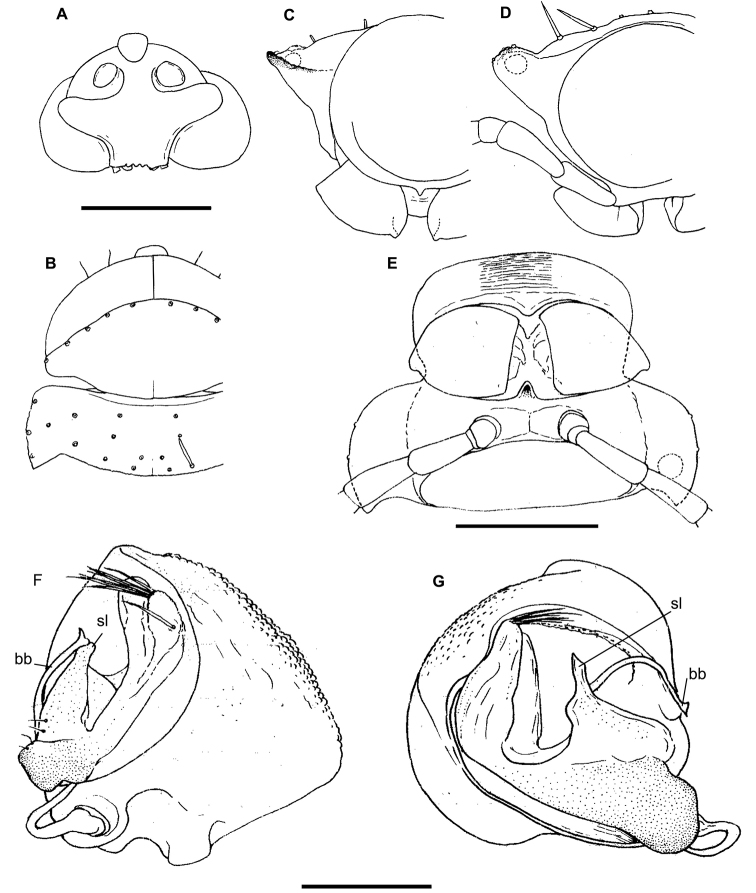
*Hemisphaeropariaintegrata* (Porat, 1894), ♂ lectotype **A** head, ventral view **B** anterior part of body, dorsal view **C, D, E** segment 7 with gonopods, oral, caudal and ventral views, respectively **F, G** right gonopod, mesal and ventromesal views, respectively. Scale bars: 0.5 mm (**A–E**), 0.1 mm (**F, G**). Figures by JPM, inked by M. Bertoncini.

Differs from other species of the genus by 20 body segments, the presence of a boletiform epicranial tubercle (♂), coupled with the gonopodal telopodites which are deeply sunken inside a large gonocoel and show only one, flagelliform, basal, main branch (bb) exposed beyond the coxa. The solenomere (sl) is short and finger-shaped (Figs 27F, G).

#### Descriptive notes.

Length of lectotype ca. 8 mm, width of midbody pro- and metazonae 0.6 and 1.1 mm (♂), respectively. Coloration in alcohol red-brown (Porat 1894).

Body with 20 segments. Pore formula normal, but slightly abbreviated: 5, 7, 9, 10, 12, 13, 15–17. Head with a distinct, epicranial, boletiform tubercle (♂)(Figure 27A, B). Interantennal isthmus ca. 1.5 times as broad as diameter of antennal socket (Figure 27A). In width, head = collum < segment 2 (Figure 27B). Paraterga relatively well-developed (Figure 27C–E), mostly slightly upturned caudally. Tarsal brushes present (♂).

Gonopodal telopodites deeply sunken inside a large gonocoel and showing only one, flagelliform, basal, main branch (bb) exposed beyond coxa; solenomere (sl) short and finger-shaped (Figs 27F, G).

**Figure 28.**
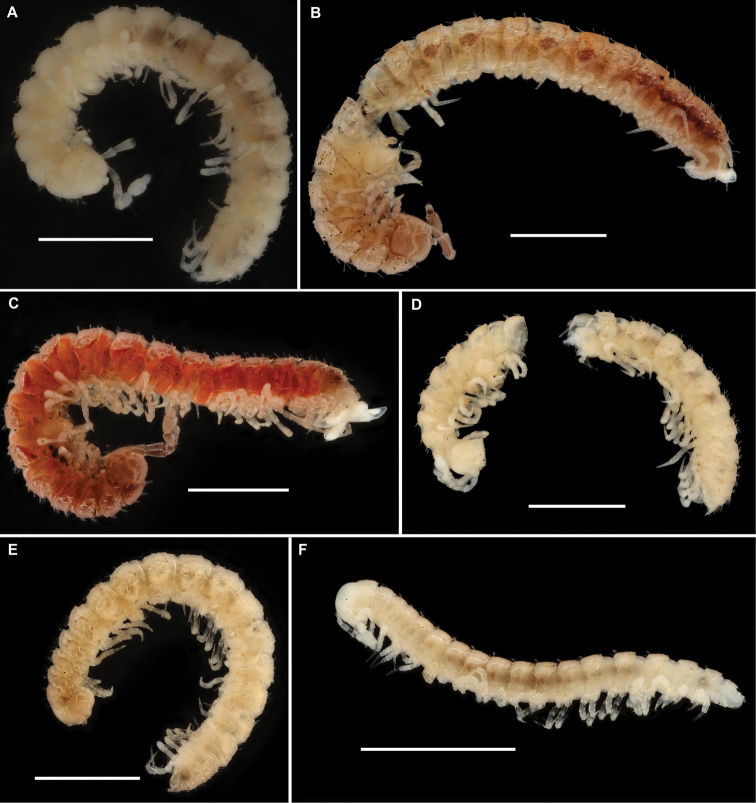
Habitus of *Hemisphaeroparia* species in lateral view, holotypes of *H.zamakoe* sp. n. (**A**), *H.bangoulap* sp. n. (**B**), *H.spiniger* sp. n. (**C**), *H.ongot* sp. n. (**D**), *H.digitifer* sp. n. (**E**) and *H.parva* sp. n. (**F**).

### Key to Trichopolydesmidae from Cameroon

The following key is proposed to separate all 13 adequately known species of the family Trichopolydesmidae recorded from Cameroon (based on male characters):

**Table:** 

1(2)	Head and collum strictly equal in width (Fig. 27B). Body with 20 segments. Only one, slender, flagelliform, main branch (basal branch, bb) of gonopodal telopodite exposed beyond coxa (Fig. 27F, G)	***H.integrata* comb. n.**
2(1)	Head always at least slightly broader than collum (e.g., Figs 3G, 5D, 9D etc.). Body with 19 or 20 segments. Usually more than one main branch of gonopodal telopodite exposed beyond coxa, but if only a single branch is exposed, then it is apical branch (ab), much stronger and non-flagelliform (e.g., Figs 14, 18, 22, 24).	**3**
3(4)	Male head without epicranial modifications.	**5**
4(3)	Male head with epicranial modifications (a central bulge or tubercle).	**7**
5(6)	Body dark, with 20 segments, 8.5–9.5 mm long (Fig. 29A). Gonopodal telopodite with three main branches (ab, mb and bb) and a lobe (lo) well-exposed beyond coxa (Fig. 16).	***H.fusca* sp. n.**
6(5)	Body light, with 19 segments, 2.8–3.2 mm long (Fig. 28F). Gonopodal telopodite with only a single strong branch (ab) well-exposed beyond coxa (Fig. 14).	***H.parva* sp. n.**
7(8)	Gonopodal telopodite with only a single main branch (ab), this being strongly exposed beyond coxa (Figs 18, 22, 24). Body with 19 or 20 segments.	**9**
8(7)	Gonopodal telopodite with 2–3 more or less strong main branches (either ab, mb and bb or ab and bb) clearly exposed beyond coxa (e.g., Figs 6, 8, 25 etc.). Body with 20 segments.	**13**
9(10)	Body with 20 segments, ca. 7 mm long (Fig. 29B). Main branch (ab) of gonopodal telopodite suberect, bifid and curved only apically (Fig. 18).	***H.bonakanda* sp. n.**
10(9)	Body with 19 segments, ca. 3–3.8 mm long. Main branch (ab) of gonopodal telopodite especially long, strong and clearly curved all along.	**11**
11(12)	Male epicranial tubercle with a tight bunch of filaments directed forward (Fig. 23L). Gonopodal telopodite with a particularly long and strongly curved apical branch (ab)(Fig. 24).	***H.falcata* sp. n.**
12(11)	Male epicranial tubercle boletiform, devoid of filaments (Fig. 21A, D, G). Gonopodal telopodite with a shorter and less strongly curved apical branch (ab)(Fig. 22).	***H.subfalcata* sp. n.**
13(14)	Gonopodal telopodite with three main branches (ab, mb and bb)(Figs 10, 12).	**15**
14(13)	Gonopodal telopodite with only two main branches (ab and bb), sometimes followed by a lobe (lo) more basally (e.g., Figs 4, 6, 8 etc.).	**17**
15(16)	Caudal corner of paraterga always rounded, drawn increasingly back, but reaching beyond rear tergal margin on segments 13–18 (Figs 9A–C). Main branch bb of gonopodal telopodite the shortest, branch ab the longest and spiniform, while a solenomere (sl) present and supplied with a tooth (t) near base (Fig. 10).	***H.ongot* sp. n.**
16(15)	Caudal corner of paraterga always rounded, drawn increasingly back, but reaching beyond rear tergal margin only on segments 17 and 18 (Figs 9A, C). Main branch bb of gonopodal telopodite the longest and distally curved, branch ab the largest, a solenomere missing, while a prominent setose finger (d) present in apical part (Fig. 12).	***H.digitifer* sp. n.**
17(18)	Male head with a large, round, micropilose bulge (Fig. 19G). Main branch ab of gonopodal telopodite the longest, main branch bb shorter, subtruncate, with a large tooth (t) distally (Fig. 20).	***H.bamboutos* sp. n.**
18(17)	Male head with a boletiform epicranial tubercle. Shapes and proportions of ab and bb branches otherwise.	19
19(20)	A vestigial solenomere (sl) with a conspicuous rod (t) near base, while a prominent lobe (lo) with a remarkable thick-walled gutter (g) laterally at base (Figure 6).	***H.bangoulap* sp. n.**
20(19)	Solenomere (sl) evident, neither a process nor a tooth at its base, nor a lateral gutter at base of lobe (lo).	**21**
21(22)	Two long setae at base of gonopodal coxa particularly strong. Both main branches of gonopodal telopodite different in length, bb being clearly curved at tip and shorter than a larger and more complex ab (Fig. 26).	***H.mouanko* sp. n.**
22(21)	Two long setae at base of gonopodal coxa relatively less strong. Both main branches of gonopodal telopodite slender and either subequal in length or bb slightly longer than ab.	**23**
23(24)	Gonopodal telopodite with a conspicuous, fully concealed, apical spine (sp), both ab and bb branches subequally long (Fig. 8).	***H.spiniger* sp. n.**
24(23)	Gonopodal telopodite without an apical spine, branch ab considerably larger, but shorter than a flagelliform bb (Fig. 4).	***H.zamakoe* sp. n.**

**Figure 29.**
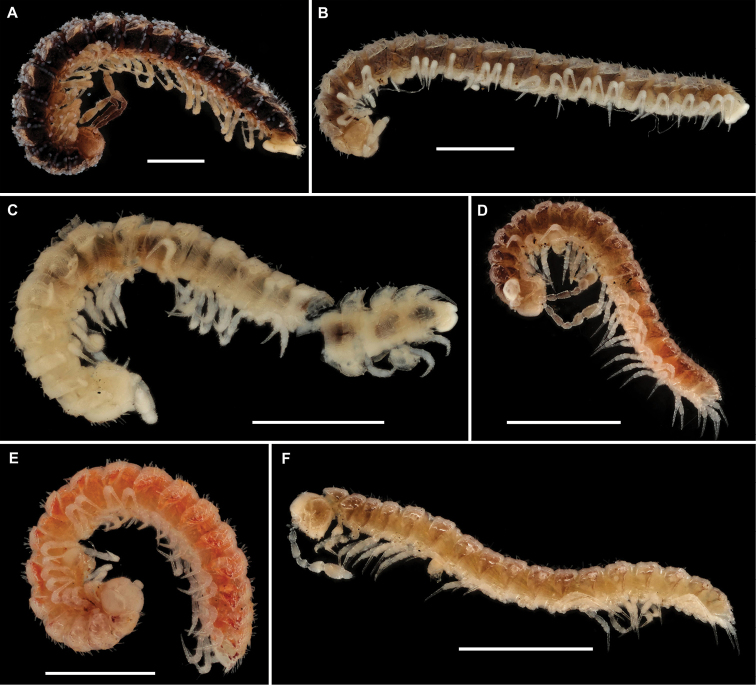
Habitus of *Hemisphaeroparia* species in lateral view, holotypes of *H.fusca* sp. n. (**A**), *H.bonakanda* sp. n. (**B**), *H.bamboutos* sp. n. (**C**), *H.subfalcata* sp. n. (**D**), *H.falcata* sp. n. (**E**) and *H.mouanko* sp. n. (**F**).

## Conclusions

**Figure 30.**
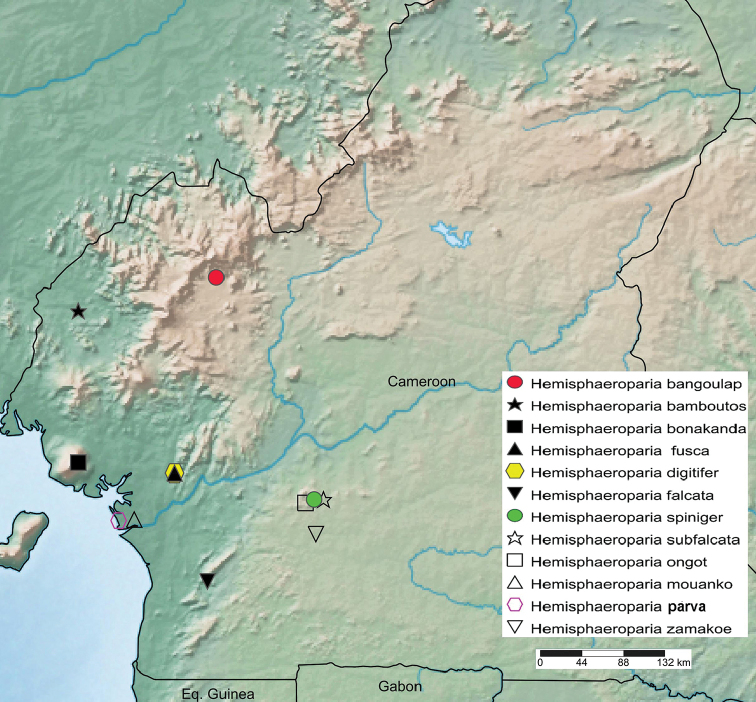
Distribution of the species of Trichopolydesmidae in Cameroon, arranged from north to south: *Hemisphaeropariabangoulap* sp. n. (**1**), *Hemisphaeropariabamboutos* sp. n. (**2**), *Hemisphaeropariabonakanda* sp. n. (**3**), *Hemisphaeropariafusca* sp. n. (**4**), *Hemisphaeropariadigitifer* sp. n. (**5**), *Hemisphaeropariafalcata* sp. n. (**6**), *Hemisphaeropariaspiniger* sp.n. (**7**), *Hemisphaeropariasubfalcata* sp. n. (**8**), *Hemisphaeropariaongot* sp. n. (**9**), *Hemisphaeropariamouanko* sp. n. (**10**), *Hemisphaeropariaparva* sp. n. (**11**), *Hemisphaeropariazamakoe* sp.n. (**12**), *Hemisphaeropariaintegrata* (Porat, 1894)(?) and ?*Hemisphaeropariaparvula* (Porat, 1894)(?).

The distribution of the genus *Hemisphaeroparia*, hitherto known to comprise a single, and type, species from Mt Nimba (Côte d’Ivoire and Guinea)(Schubart 1955), appears to presently cover much of western Africa. Furthermore, the whole of Cameroon, at least its better-studied southern half, supports species of this genus alone (Figure 30). Our contribution has enriched the entire fauna of African Trichopolydesmidae by one-third, while the fauna of Cameroon becomes the best studied across the whole continent. There is little doubt that this family is taxonomically one of the most diverse throughout Africa, with numerous further species still awaiting discovery even in Cameroon. We may say that we have just touched the tip of the iceberg.

Sympatry or even syntopy is not too rare among Afrotropical Trichopolydesmidae. Thus, Schubart (1955) described three different species (and genera) from Mt Nimba. Mauriès and Heimer (1996) not only published several species occurring sympatrically from eastern Africa, but they also provided a general map showing the distribution of the family and all of its genera and species then known on the continent. Our material likewise demonstrates a few cases of sympatry or strict syntopy in Cameroon, up to three species per locality (Figure 30).

With a list of already 14 species in *Hemisphaeroparia* alone, some of them may be grouped in a few species groups. Thus, because *H.subfalcata* sp. n. and *H.falcata* sp. n. share not only the very small body with 19 segments and certain epicranial modifications (♂), but also the particularly long branches ab of the gonopods (Figs 22, 24), they seem to belong to the same species group. Perhaps at least some of the species that show only two or all three main branches of the gonopodal telopodite form further 2–3 species groups, but we shall refrain from further outlining and naming them now pending more material becomes described. Some is already available, but remains entirely unstudied yet; further samples may be expected to come in the near future, and all this, as well as a complete species-level reclassification of African Trichopolydesmidae will be treated in the next part of our paper.

## Supplementary Material

XML Treatment for Bactrodesmus

XML Treatment for Dendrobrachypus

XML Treatment for Eburodesmus

XML Treatment for Elgonicola

XML Treatment for Hemisphaeroparia

XML Treatment for Heterosphaeroparia

XML Treatment for Mabocus

XML Treatment for Mecistoparia

XML Treatment for Megaloparia

XML Treatment for Physetoparia

XML Treatment for Sphaeroparia

XML Treatment for Trichozonus

XML Treatment for Sphaeroparia

XML Treatment for Physetoparia

XML Treatment for Mecistoparia

XML Treatment for Eburodesmus

XML Treatment for Hemisphaeroparia

XML Treatment for Hemisphaeroparia zamakoe

XML Treatment for Hemisphaeroparia bangoulap

XML Treatment for Hemisphaeroparia spiniger

XML Treatment for Hemisphaeroparia ongot

XML Treatment for Hemisphaeroparia digitifer

XML Treatment for Hemisphaeroparia parva

XML Treatment for Hemisphaeroparia fusca

XML Treatment for Hemisphaeroparia bonakanda

XML Treatment for Hemisphaeroparia bamboutos

XML Treatment for Hemisphaeroparia subfalcata

XML Treatment for Hemisphaeroparia falcata

XML Treatment for Hemisphaeroparia mouanko

XML Treatment for Hemisphaeroparia integrata

## Footnotes

Shear and Reddell (2017) have recently suggested to consider the polydesmidan gonopodal coxae fused only when the medial fusion is as strong as that observed in the Dalodesmidea. However, the gonocoxae in the Polydesmidea are also fused not only basally to their sternum, but also medially through two small chitinous flaps, each typically supporting two strong setae. However slight, this is also fusion, a condition which may be termed “narrowly fused”.
